# Asymmetric Electrophilic Difluoromethylthiolation of Indanone-Based β-Keto Esters Using Difluoromethanesulfonyl Hypervalent Iodonium Ylides

**DOI:** 10.3390/molecules24020221

**Published:** 2019-01-09

**Authors:** Satoshi Gondo, Okiya Matsubara, Hélène Chachignon, Yuji Sumii, Dominique Cahard, Norio Shibata

**Affiliations:** 1 Department of Nanopharmaceutical Sciences, Department of Life Science and Applied Chemistry, Nagoya Institute of Technology, Gokiso, Showa-ku, Nagoya 466-8555, Japan; 29411074@stn.nitech.ac.jp (S.G.); cjh11141@nitech.jp (O.M.); sumii.yuji@nitech.ac.jp (Y.S.); 2 CNRS, UMR 6014 COBRA, Normandie Université, 1 Rue Tesnière, F-76821 Mont-Saint-Aignan Cedex, France; helene.chachignon@insa-rouen.fr (H.C.); dominique.cahard@univ-rouen.fr (D.C.); 3 Institute of Advanced Fluorine-Containing Materials, Zhejiang Normal University, 688 Yingbin Avenue, Jinhua 321004, China

**Keywords:** fluorine, sulfur, asymmetric synthesis, hypervalent iodine

## Abstract

The first electrophilic diastereoselective direct introduction of the difluoromethylthio group is described. We used a chiral auxiliary-based approach to illustrate the versatility of our recently developed difluoromethanesulfonyl hypervalent iodonium ylide reagents for the difluoromethylthiolation of indanone-based β-keto esters. Chiral SCF_2_H-featuring compounds were obtained in up to 93% ee value.

## 1. Introduction

**Scheme 1:**
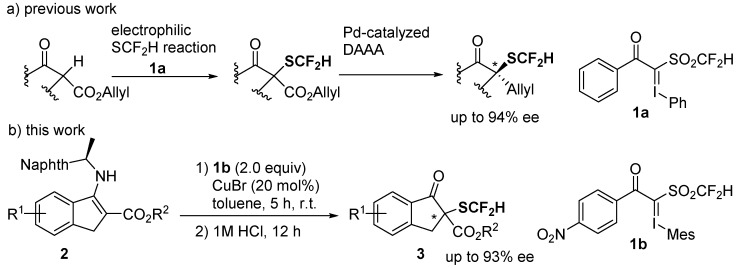
Two methods for the asymmetric synthesis of difluoromethylthio-compounds with a tetrasubstituted carbon center; (**a**) previous work; (**b**) this work. The asterisk indicates the chiral center in the molecules.

In the field of organofluorine chemistry one of the major present concerns is the development of new methods for the construction of novel chemical scaffolds. In this vein, the combination of sulfur, carbon and fluorine atoms has given birth to emergent motifs, which include SCF_3_, SCF_2_H, SCF_2_FG (FG = SO_2_Ar, SAr, PO(OR)_2_, COAr, Rf), SCFH_2_ [1,2,3,4]. The most recurring motif is undoubtedly the SCF_3_ one, which use grew at an unprecedented rate in the past recent years. The SCF_3_ chemotype is encountered in several biologically active molecules, albeit virtually absent in marketed drugs. The reason it elicits such enthusiasm is the exceptional high lipophilicity of SCF_3_ molecules that confers a high potential in medicinal chemistry. Equally interesting, though less often investigated, is the SCF_2_H group that also possess high lipophilicity while acting as hydrogen-bond donor owing to the acidity of the hydrogen atom [5]. The synthesis of enantioenriched molecules featuring a SCF_2_R motif directly linked to the chiral center is an issue worth consideration in the context of designing new chiral drugs. Asymmetric synthesis of trifluoromethylthiolated compounds have been investigated [6,7,8,9,10,11,12,13,14,15,16,17,18,19,20,21,22], and very recently Shibata and co-workers published the asymmetric synthesis of α-tri- and difluoromethylthio allyl ketones via electrophilic difluoromethylthiolation of β-keto esters using difluoromethanesulfonyl hypervalent iodonium ylide **1a** followed by a Pd-catalyzed Tsuji decarboxylative asymmetric allylic alkylation (DAAA, Scheme 1a) [23]. However, there is no report describing the asymmetric synthesis of difluoromethylthio compounds via a direct difluoro- methylthiolation reaction. Hence, we have been interested in direct asymmetric electrophilic difluoromethylthiolations. For this purpose, we targeted SCF_2_H analogues of α-hydroxy β-keto esters, in particular those with an indanone scaffold [24,25,26] that are ubiquitous and important structural motifs, as such or in a masked form, in a wide range of biologically active natural products and synthetic pharmaceuticals and agrochemicals [27,28,29,30,31,32,33,34]. Moreover, we decided to valorize our difluoromethanesulfonyl hypervalent iodonium ylides **1a**,**b** as electrophilic difluoromethyl- thiolation reagents for a wide range of nucleophiles [24]. Herein, we report the first asymmetric electrophilic introduction of the difluoromethylthio group onto chiral enamines derived from β-keto esters (Scheme 1b).

## 2. Results and Discussion

We recently demonstrated that the difluoromethylthiolation of enamines obtained from β-keto esters was efficient and had wide generality [24], thus we surmised that the enamine approach would nicely extend to chiral enamines. In a first series of experiments, we studied the difluoro- methylthiolation of β-enamino esters **2a**–**e** prepared from methyl 6-methyl-1-indanone-2- carboxylate and various chiral amines in order to determine the most appropriate chiral auxiliary (Table 1). The optimized reaction conditions found for the difluoromethylthiolation of achiral β-enamino esters were first applied to chiral β-enamino esters **2a**–**e**. In the presence of 20 mol% of copper bromide, the difluoromethanesulfonyl hypervalent iodonium ylide **1b** reacted in 1,4-dioxane at room temperature for 5 h followed by acidic cleavage of the resulting imine product to afford the 2-difluoromethylthio-1,3-dicarbonyl compound **3**. The (*S*)-(–)-α-methylbenzylamine auxiliary gave the desired product **3** in good yield and an encouraging 57% ee value (Table 1, entry 1). Variations of the Ar and R groups of the chiral amine indicated that the bulkier (*S*)-(–)-1-(1-naphthyl)ethylamine led to enhanced enantioselectivity (Table 1, entry 3) albeit in a lower chemical yield.

Next, we conducted a second series of experiments in order to optimize the reaction solvent with substrates **2a** or **2c**. Solvent screening revealed an increase in enantioselectivity in going from ether-containing solvents (1,4-dioxane, THF), chlorinated solvents (CH_2_Cl_2_, CHCl_3_) to aromatic toluene, which provided the highest ee values of 69 and 88% ee, respectively, for both phenyl and naphthyl-based auxiliaries (Table 2). A survey of other parameters that are the amount and the nature (**1a** versus **1b**) of the difluoromethanesulfenylating reagent and the amount and nature of the copper catalyst was also performed but deviation from standard conditions did not allow to improve the reactivity nor the enantioselectivity. We further attempted the reaction of β-keto esters with **1b** in the presence of a catalytic amount of chiral amine, (*R*)-1-(naphthalen-1-yl)ethan-1-amine, but the reaction did not proceed well giving **3a** in a low yield (<10%).

**Scheme 2:**
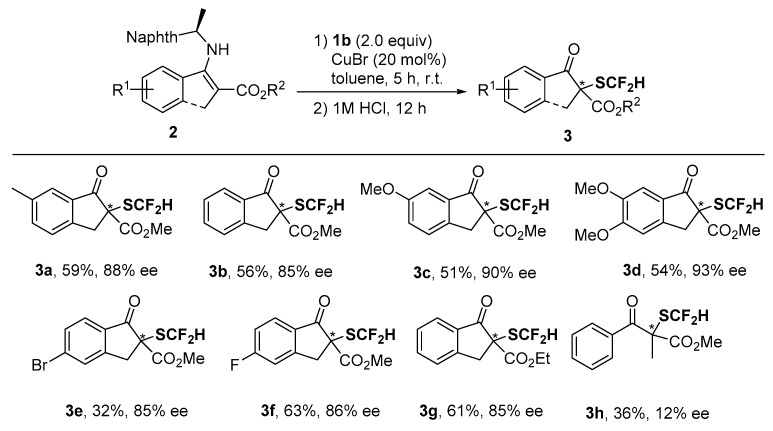
Difluoromethylthiolation of various indanone-based enamino esters and one acyclic enamino ester. The asterisk indicates the chiral center in the molecules **3**.

Having identified the suitable chiral auxiliary and the reaction conditions, we then turned our efforts to exploring other indanone-based enamine substrates featuring various substituents on the aromatic ring (Scheme 2).

We noticed that the enantiomeric excess increased for electron-donating substituents (MeO > Me > H) with a cumulative effect (two MeO > MeO, products **3a**–**d**). Halogen substituted indanone-based enamines were compatible with the reaction conditions and gave similar ee values to the undecorated indanone (**3e**,**f** versus **3b**). The size of ester does not much affect the yield and enantioselectivity on the transformation (**3b**, R^2^ = Me and **3g**, R^2^ = Et, Scheme 2). In addition to these indanone carboxylates, we also attempted the substrates having six-membered tetralone-type structure and acyclic substrates. However, the tetralone-type substrate failed to deliver the corresponding β-enamino ester and an acyclic β-enamino ester produced a SCF_2_H-product **3h** with a low ee (12%, see Scheme 2). The chiral amine auxiliary was recovered in 25% yield after the reaction with **2d** (not optimized) [36].

**Scheme 3:**
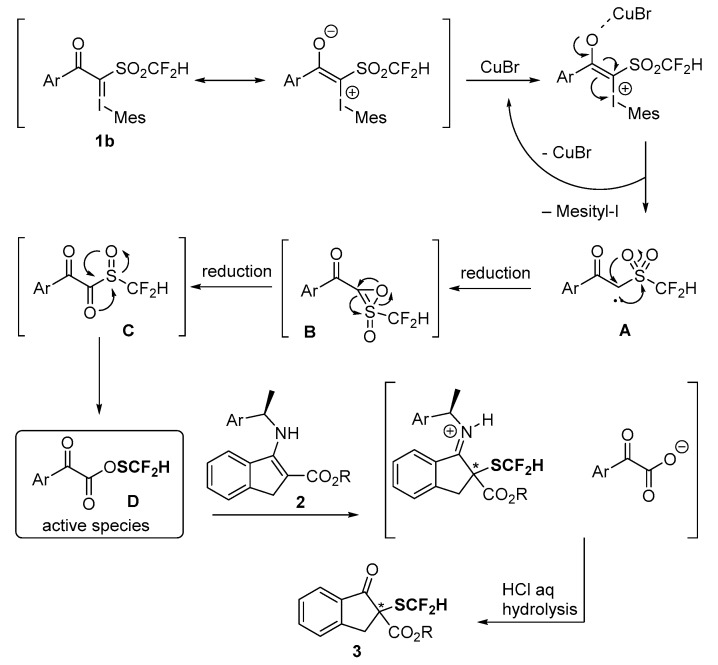
Proposed reaction mechanism. The asterisk indicates the chiral center.

With regard to the reaction mechanism based on our previous reports [24,37], we proposed a copper-catalyzed generation of carbene **A** by reaction of the difluoromethanesulfonyl hypervalent iodonium ylide **1b** followed by formation of the oxathiirene-2-oxide **B**, which rearranged to the sulfoxide **C** and collapsed into the thioperoxoate **D**. This SCF_2_H thioperoxoate was supposed to be the active electrophilic HF_2_CS^+^ donor that reacted with the β-enamino esters **2**. The resulting iminium was then hydrolyzed under acidic conditions to release the desired α-SCF_2_H β-keto esters **3** (Scheme 3).

## 3. Materials and Methods

### 3.1. General Information

All reagents were used as received from commercial sources, unless specified otherwise. Enamino esters were prepared referring to previously reported procedures [37,38,39]. Reactions requiring anhydrous conditions were performed in flame-dried glassware under a positive pressure of nitrogen. Reaction mixtures were stirred magnetically. Solvents were transferred via syringe and were introduced into the reaction vessels though a rubber septum. All of the reactions were monitored by thin-layer chromatography (TLC) carried out on 0.25 mm silica-gel (60-F_254_) (Merck, Kenilworth, NJ, USA). The TLC plates were visualized with UV light and 7% phosphomolybdic acid or KMnO_4_ in water/heat. Preparative thin-layer plates carried out on 2.0 mm Merck silica gel (60-F_254_). Column chromatography was carried out on a column packed with silica-gel 60N spherical neutral size 50–63 μm. The ^1^H-NMR (300, 700 MHz) was recorded on a Varian Mercury 300 (Agilent Technologies, Palo Alto, CA, USA) or an ECZ-700R (JEOL Ltd, Tokyo, Japan) instrument, with TMS (δ = 0.00 ppm) as internal standard, and ^19^F-NMR (282 MHz) spectra was recorded on a Varian Mercury 300 with C_6_F_6_ (δ = −162.2 ppm) as internal standard. The ^13^C-NMR (125 MHz) spectra were recorded on an Avance 500 spectrometer (Bruker, Billerica, MA, USA). Chemical shifts (δ) are reported in parts per million and coupling constants (*J*) are in hertz. The following abbreviations were used to show the multiplicities: s: singlet, d: doublet, t: triplet, q: quadruplet, dd: doublet of doublets, td: triplet of doublets, dt: doublet of triplets, m: multiplet, br: broad. All the melting points are uncorrected. Mass spectra were recorded on an LCMS-2020EV (ESI-MS) system (Shimadzu Corporation, Kyoto, Japan). Infrared spectra were recorded on a FT/IR-4100 spectrometer (JASCO Corporation, Tokyo, Japan). HPLC analyses were performed on a JASCO PU-2080 Plus system using a 4.6 × 250 mm CHIRALPAK IB-3 column and a CHIRALCEL OD-3 column. Optical rotations were measured on a SEPA-300 instrument (HORIBA Ltd, Kyoto, Japan). High resolution mass spectrometry were recorded on a Synapt G2 HDMS (ESI-MS) system (Waters Corporation, Milford, MA, USA). The chiral amines: (*S*)-(−)-α-methylbenzylamine (≥99.5% ee), (*S*)-(−)-α-ethylbenzylamine (≥99.0% ee), (*S*)-(−)-4-methoxy- α-methylbenzylamine (≥97.5% ee) were purchased from Sigma Aldrich (St. Louis, MI, USA). (*S*)-(–)-1-(1-Naphthyl)ethylamine (>98.0% ee), and (*S*)-(−)-1-(*p*-tolyl)ethylamine were purchased from TCI (Tokyo, Japan). The ^1^H, ^13^C and ^19^F NMR spectra of compounds **3** and HPLC data of compounds **3** are available in the Supplementary Material.

### 3.2. Synthesis of Chiral Enamine (General Procedure)

Amine (2.2 mmol, 1.5 equiv) was added to a solution of β-ketoester (1.47 mmol) and zinc acetate (0.29 mmol, 20 mol%) in methanol under nitrogen atmosphere, the reaction mixture was refluxed for 16–64 h. After the reaction, the mixture was concentrated under reduced pressure, and the residue was purified by column chromatography (with ethyl acetate: hexane mixtures as eluent).

*Methyl 5-methyl-3-((1-phenylethyl)amino)-1H-indene-2-carboxylate* (**2a**). Following the general procedure the reaction mixture was stirred at 50 °C for 53 h. After the reaction was complete, the mixture was worked up as described. Brown solid (35%, 156.6 mg). ^1^H-NMR (300 MHz, CDCl_3_) δ 8.29 (d, *J* = 6.3 Hz, 1H), 7.21–7.43 (m, 7H), 7.11 (d, *J* = 7.8 Hz, 1H), 5.36–5.41 (m, 1H), 3.81 (s, 3H), 3.50 (s, 2H), 2.27 (s, 3H), 1.66 (d, *J* = 6.8 Hz, 3H). ^13^C-NMR (125 MHz, CDCl_3_) δ 21.5, 26.0, 33.9, 50.4, 53.8, 97.2, 124.3, 124.5, 125.4 (2C), 127.0, 128.7 (2C), 129.2, 135.6, 137.6, 142.6, 145.3, 159.1, 168.8. IR (KBr): 3284, 2958, 2924, 1643, 1587, 1564, 1444, 1317, 1267, 1205 cm^−1^. MS (ESI): *m*/*z* 308 (M + H)^+^.

*Methyl 5-methyl-3-((1-phenylpropyl)amino)-1H-indene-2-carboxylate* (**2b**). Gray solid (30%, 153.5 mg). ^1^H-NMR (300 MHz, CDCl_3_) δ 8.36 (d, *J* = 8.1 Hz, 1H), 7.19–7.40 (m, 7H), 7.10 (d, *J* = 7.8 Hz, 1H), 5.09–5.15 (m, 1H), 3.82 (s, 3H), 3.49 (s, 2H), 2.27 (s, 3H), 1.90–1.99 (m, 2H), 1.06 (t, *J* = 7.1 Hz, 3H). ^13^C-NMR (125 MHz, CDCl_3_) δ 10.7, 21.5, 32.8, 33.9, 50.3, 59.8, 97.1, 124.3, 124.5, 126.0 (2C), 126.9, 128.6 (2C), 129.2, 135.5, 137.6, 142.6, 144.0, 159.6, 168.9. IR (KBr): 3273, 2970, 2951, 1651, 1595, 1568, 1460, 1309, 1263, 1194 cm^−1^. MS (ESI): *m*/*z* 322 (M + H)^+^.

*Methyl 5-methyl-3-((1-(naphthalen-1-yl)ethyl)amino)-1H-indene-2-carboxylate* (**2c**). Pale yellow solid (40%, 211.0 mg). ^1^H-NMR (300 MHz, CDCl_3_) δ 8.48 (d, *J* = 6.3 Hz, 1H), 8.16 (d, *J* = 8.4 Hz, 1H), 7.89 (d, *J* = 8.1 Hz, 1H), 7.71 (d, *J* = 8.4 Hz, 1H), 7.60–7.66 (m, 2H), 7.55–7.50 (m, 1H), δ 7.38 (t, *J* = 7.7 Hz, 1H), 7.20–7.24 (m, 1H), 7.10 (s, 1H), 6.97 (d, *J* = 7.5 Hz, 1H), 6.09–6.14 (m, 1H), 3.83 (s, 3H), 3.49 (d-like, 2H), 1.83 (s, 3H), 1.78 (d, *J* = 6.6 Hz, 3H). ^13^C-NMR (125 MHz, CDCl_3_) δ 21.0, 24.8, 33.9, 50.1, 50.4, 96.8, 121.7, 122.6, 124.1, 124.3, 125.5, 125.9, 126.4, 127.6, 129.1, 129.2, 129.7, 133.8, 135.3, 137.4, 140.9, 142.4, 159.0, 168.9. IR (KBr): 3296, 3059, 2885, 2862, 1655, 1591, 1564, 1448, 1267, 1186 cm^−1^. MS (ESI): *m*/*z* 358 (M + H)^+^.

*Methyl 5-methyl-3-((1-(p-tolyl)ethyl)amino)-1H-indene-2-carboxylate* (**2d**). Yellow solid (20%, 96.6 mg). ^1^H-NMR (300 MHz, CDCl_3_) δ 8.26 (d, *J* = 6.9 Hz, 1H), 7.41 (s, 1H), 7.28–7.29 (m, 3H), 7.09–7.14 (m, 3H), 5.35 (t, *J* = 6.6 Hz, 1H), 3.80 (s, 3H), 3.48 (s, 2H), 2.30 (s, 3H), 2.28 (s, 3H), 1.64 (d, *J* = 3.6 Hz, 3H). ^13^C-NMR (125 MHz, CDCl_3_) δ 21.0, 21.6, 26.1, 33.9, 50.4, 53.5, 97.0, 124.4, 124.5, 125.3 (2C), 129.2, 129.4 (2C), 135.6, 136.5, 137.6, 142.3, 142.7, 159.2, 168.9. IR (KBr): 3307, 3032, 2924, 2316, 1651, 1595, 1556, 1448, 1263, 1201 cm^−1^. MS (ESI): *m*/*z* 322 (M + H)^+^.

*Methyl 3-((1-(4-methoxyphenyl)ethyl)amino)-5-methyl-1H-indene-2-carboxylate* (**2e**). Pale yellow solid (32%, 156.3 mg). ^1^H-NMR (300 MHz, CDCl_3_) δ 8.23 (d, *J* = 7.2 Hz, 1H), 7.40 (s, 1H), 7.25–7.34 (m, 3H), 7.11 (d, *J* = 7.2 Hz, 1H), 6.86 (d, *J* = 8.4 Hz, 2H), 5.31–5.36 (m, 1H), 3.79 (s, 3H), 3.76 (s, 3H), 3.48 (s, 2H), 2.28 (s, 3H), 1.63 (d, *J* = 6.3 Hz, 3H). ^13^C-NMR (125 MHz, CDCl_3_). IR (KBr): 3276, 2997, 2939, 2831, 1647, 1610, 1587, 1506, 1452, 1329, 1259, 1190, 1174, 1092 cm^−1^. MS (ESI): *m*/*z* 338 (M + H)^+^.

*Methyl 3-((1-(naphthalen-1-yl)ethyl)amino)-1H-indene-2-carboxylate* (**2f**). Yellow solid (42%, 214.1 mg). ^1^H-NMR (300 MHz, CDCl_3_) δ 8.47 (d, *J* = 6.6 Hz, 1H), 8.14 (d, *J* = 8.4 Hz, 1H), 7.92 (d, *J* = 8.1 Hz, 1H), 7.74 (d, *J* = 8.4 Hz, 1H), 7.52–7.67 (m, 3H), 7.37–7.42 (m, 2H), 7.29 (d, *J* = 7.8 Hz, 1H), 7.20 (t, *J* = 7.5 Hz, 1H), 6.91 (t, *J* = 7.7 Hz, 1H), 6.07–6.17 (m, 1H), 3.84 (s, 3H), 3.56 (s, 2H), 1.79 (d, *J* = 6.6 Hz, 3H). ^13^C-NMR (125 MHz, CDCl_3_) δ 24.9 34.3, 50.5, 96.7, 122.0, 122.5, 123.3, 124.9, 125.6, 125.9, 126.3, 126.5, 127.7, 128.2, 129.3, 129.8, 134.0, 137.3, 140.6, 145.5, 159.0, 169.0. IR (KBr): 3292, 3062, 2966, 2951, 1747, 1655, 1606, 1568, 1444, 1529, 1190 cm^−1^. MS (ESI): *m*/*z* 344 (M + H)^+^.

*Methyl 5-methoxy-3-((1-(naphthalen-1-yl)ethyl)amino)-1H-indene-2-carboxylate* (**2g**). Yellow solid (46%, 250.4 mg). ^1^H-NMR (300 MHz, CDCl_3_) δ 8.48 (d, *J* = 4.5 Hz, 1H), 8.16 (d, *J* = 8.1 Hz, 1H), 7.91 (d, *J* = 7.8 Hz, 1H), 7.70–7.76 (m, 2H), 7.50–7.63 (m, 2H), 7.42 (t, *J* = 7.5 Hz, 1H), 7.21 (d, *J* = 8.7 Hz, 1H), 6.73 (dd, *J* = 8.4, 2.4 Hz, 1H), 6.58 (d, *J* = 2.4 Hz, 1H), 6.01–6.09 (m, 1H), 3.85 (s, 3H), 3.53 (d, *J* = 22.2 Hz, 1H), 3.44 (d, *J* = 22.2 Hz, 1H), 2.71 (s, 3H), 1.82 (d, *J* = 6.9 Hz, 3H). ^13^C-NMR (125 MHz, CDCl_3_) δ 33.6, 50.5, 50.6, 54.3, 97.7, 106.6, 116.9, 121.9, 122.7, 125.2, 125.7, 126.2, 126.5, 127.6, 129.3, 129.6, 134.0, 137.6, 138.1, 141.0, 158.1, 159.1, 168.9. IR (KBr): 3300, 3057, 2945, 2829, 1741, 1655, 1614, 1576, 1452, 1225, 1132, 1086 cm^−1^. MS (ESI): *m*/*z* 374 (M + H)^+^.

*Methyl 5,6-dimethoxy-3-((1-(naphthalen-1-yl)ethyl)amino)-1H-indene-2-carboxylate* (**2h**). Yellow solid (45%, 265.6 mg). ^1^H-NMR (300 MHz, CDCl_3_) δ 8.54 (br s, 1H), δ 8.17 (d, *J* = 8.7 Hz, 1H), 7.91 (d, *J* = 8.1 Hz, 1H), 7.69–7.75 (m, 2H), 7.50–7.63 (m, 2H), 7.38–7.43 (m, 1H), 6.85 (s, 1H), 6.47 (s, 1H), 5.96–6.05 (m, 1H), 3.84 (s, 3H), 3.80 (s, 3H), 3.53 (d, *J* = 22.2 Hz, 1H), 3.44 (d, *J* = 22.2 Hz, 1H) 2.61 (s, 3H), 1.83 (d, *J* = 6.3 Hz, 3H). ^13^C-NMR (125 MHz, CDCl_3_) δ 24.7, 34.1, 50.3, 50.5, 54.6, 55.8, 95.3, 106.0, 107.2, 121.8, 122.7, 125.7 126.2, 126.5, 127.6, 129.1, 129.4, 129.5, 133.9, 139.1, 140.9, 147.4, 149.8, 159.7, 168.6. IR (KBr): 3300, 2947, 1739, 1643, 1595, 1556, 1448, 1309, 1252, 1209 cm^−1^. MS (ESI): *m*/*z* 404 (M + H)^+^.

*Methyl 6-bromo-3-((1-(naphthalen-1-yl)ethyl)amino)-1H-indene-2-carboxylate* (**2i**). Yellow solid (42%, 262.9 mg). ^1^H-NMR (300 MHz, CDCl_3_) δ 8.41 (br d, 1H), 8.10 (d, *J* = 8.7 Hz, 1H), 7.92 (d, *J* = 8.1 Hz, 1H), 7.74 (d, *J* = 7.5 Hz, 1H), 7.50–7.65 (m, 4H), 7.39 (t, *J* = 7.8 Hz, 1H), 7.09 (d, *J* = 8.4 Hz, 1H), 7.01 (d, *J* = 8.4 Hz, 1H), 6.00–6.05 (m, 1H), 3.84 (s, 3H), 3.53 (s, 2H), 1.78 (d, *J* = 6.6 Hz, 3H). ^13^C-NMR (125 MHz, CDCl_3_) δ 24.8, 34.2, 50.4, 50.6, 96.9, 121.7, 122.5, 122.8, 124.3, 125.7, 125.9, 126.7, 127.8, 128.0, 123.0, 129.4, 129.6, 134.0, 136.3, 140.3, 147.3, 158.0, 168.7. IR (KBr): 3296, 3059, 2939, 2858, 1739, 1658, 1610, 1560, 1452, 1325, 1255, 1186 cm^−1^. MS (ESI): *m*/*z* 422 (M + H)^+^.

*Methyl 6-fluoro-3-((1-(naphthalen-1-yl)ethyl)amino)-1H-indene-2-carboxylate* (**2j**). Yellow solid (43%, 230.9 mg). ^1^H-NMR (300 MHz, CDCl_3_) δ 8.47 (d, *J* = 6.9 Hz, 1H), 8.12 (d, *J* = 8.4 Hz, 1H), 7.92 (d, *J* = 8.1 Hz, 1H), 7.74 (d, *J* = 8.1 Hz, 1H), 7.60–7.66 (m, 2H), 7.53–7.58 (m, 1H), 7.37–7.42 (m, 1H), 7.19 (dd, *J* = 8.7, 5.1 Hz, 1H), 7.06 (dd, *J* = 8.4, 2.4 Hz, 1H), 6.59 (td, *J* = 9.0, 2.4 Hz, 1H), 6.00–6.09 (m, 1H), 3.84 (s, 3H), 3.59 (d, *J* = 22.8 Hz, 1H), 3.50 (d, *J* = 22.8 Hz, 1H) 1.78 (d, *J* = 6.6 Hz, 3H). ^19^F-NMR (282 MHz, CDCl_3_) δ −113.3 (s, 1F). ^13^C-NMR (125 MHz, CDCl_3_) δ 24.9, 34.4, 50.4, 50.5, 96.5, 112.1 (d, *J* = 22.5 Hz, 1C), 113.6 (d, *J* = 22.5 Hz, 1C), 121.8, 122.5,124.4 (d, *J* = 8.8 Hz, 1C), 125.7, 125.9, 126.7, 127.8, 129.4, 129.7, 133.4, 134.0, 140.4, 148.2 (d, *J* = 8.8 Hz, 1C), 158.2, 163.1 (d, *J* = 247.5 Hz, 1C), 168.8. IR (KBr): 3296, 3057, 2945, 1741, 1655, 1614, 1576, 1452, 1225, 1132, 1086 cm^−1^. MS (ESI): *m*/*z* 362 (M + H)^+^.

*Ethyl 3-((1-(Naphthalen-1-Yl)ethyl)amino)-1H-Indene-2-Carboxylate* (**2k**). Amine (2.0 mmol, 1.5 equiv) was added to a solution of β-ketoester (1.35 mmol) and zinc acetate (0.27 mmol, 20 mol%) in methanol under a nitrogen atmosphere, the reaction mixture was refluxed for 60 h. After reaction, the mixture was concentrated under reduced pressure, and the residue was purified by column chromatography (ethyl acetate: hexane). Yellow solid (43%, 205.6 mg). ^1^H-NMR (300 MHz, CDCl_3_) δ 8.45 (d, *J* = 6.0 Hz, 1H), 8.14 (d, *J* = 8.4 Hz, 1H), 7.91 (d, *J* = 7.8 Hz, 1H), 7.74 (d, *J* = 7.2 Hz, 1H), 7.52–7.68 (m, 3H), 7.37–7.42 (m, 2H), 7.29 (d, *J* = 8.1 Hz, 1H), 7.19 (t, *J* = 7.5 Hz, 1H), 6.90 (t, *J* = 7.8 Hz, 1H), 6.08–6.16 (m, 1H), 4.31 (q, *J* = 7.1 Hz, 2H), 3.61 (d, *J* = 22.8 Hz, 1H), 3.53 (d, *J* = 23.1 Hz, 1H), 1.78 (d, *J* = 6.9 Hz, 3H), 1.38 (t, *J* = 7.2 Hz, 3H). ^13^C-NMR (125 MHz, CDCl_3_) δ 14.8, 24.9, 34.4, 50.3, 58.9, 97.1, 122.0, 122.5, 123.8, 124.8, 125.5, 125.9, 126.2, 126.5, 127.6, 128.1, 129.2, 129.8, 133.9, 137.4, 140.6, 145.6, 158.7, 168.6. IR (KBr): 3296, 3059, 2974, 1739, 1647, 1610, 1591, 1564, 1441, 1259, 1186, 1120 cm^−1^. MS (ESI): *m*/*z* 358 (M + H)^+^.

### 3.3. Representative Procedure for the Diastereoselective Difluoromethylthiolation

#### General Procedure

Reagent **1b** [24] (0.40 mmol, 2.0 equiv) was added to a solution of enamine (0.20 mmol, 1.0 equiv) and CuBr (0.04 mmol, 20 mol%) in toluene (2.5 mL) under a nitrogen atmosphere, and the reaction mixture was stirred at room temperature for 5 h. HCl (1 M) was added to the reaction mixture which was then stirred for 12 h. After that, the mixture was extracted with ethyl acetate two times, then washed with brine and dried by Na_2_SO_4_. The ethyl acetate was removed under reduced pressure and the residue was purified by column chromatography (ethyl acetate: hexane) or (CH_2_Cl_2_: hexane).

*Methyl 2-((difluoromethyl)thio)-6-methyl-1-oxo-2,3-dihydro-1H-indene-2-carboxylate* (**3a**) [23,24,25]. Yellow oil (24.2 mg, 45%, 88% ee). The ee value was determined by HPLC analysis using a Chiralpack IB3 column (hexane/*i*PrOH = 98:2, flow rate: 0.5 mL/min, t_R_ (minor) =18.8 min (integral = 5.9%), t_R_ (major) = 21.8 min (integral = 94.1%). ^1^H-NMR (300 MHz, CDCl_3_) δ 7.64 (s, 1H), 7.52 (S, 1H), 7.50 (t, *J* = 55.7 Hz 1H), 7.36 (d, *J* = 7.6 Hz, 1H), 3.98 (d, *J* = 17.6 Hz, 1H), 3.81 (s, 3H), 3.22 (d, *J* = 17.9 Hz, 1H), 2.43 (s, 3H). ^19^F-NMR (282 MHz, CDCl_3_) δ −92.1 (dd, *J* = 251.7, 55.2 Hz, 1F), −93.5 (dd, *J* = 250.9, 56.0 Hz, 1F). HRMS (ESI) *m*/*z* Calcd: 309.0373 for C_13_H_12_O_3_F_2_SNa (M + Na)^+^ Found: 309.0370.

*Methyl 2-((difluoromethyl)thio)-1-oxo-2,3-dihydro-1H-indene-2-carboxylate* (**3b**). Pale yellow solid (30.7 mg, 56%, 85% ee). [α]^25^_D_ = + 6.6 (*c* = 0.77, CHCl_3_). The ee value was determined by HPLC analysis using a Chiralpack IB3 column (hexane/*i*PrOH = 98:2, flow rate: 0.5 mL/min, t_R_ (minor) = 30.2 min (integral = 7.4%), t_R_ (major) = 33.5 min (integral = 92.5%). ^1^H-NMR (700 MHz, CDCl_3_) δ 7.84 (d, *J* = 7.6 Hz, 1H), 7.70 (d, *J* = 7.6 Hz, 1H), 7.49 (t, *J* = 52.2 Hz, 1H), 7.47 (s, 3H), 7.44–7.50 (m, 2H), 4.04 (d, *J* = 17.9 Hz, 1H), 3.82 (s, 3H), 3.27 (d, *J* = 17.9 Hz, 1H). ^19^F-NMR (282 MHz, CDCl_3_) δ −92.0 (dd, *J* = 250.9, 56.0 Hz, 1F), −93.4 (dd, *J* = 250.0, 55.2 Hz, 1F). ^13^C-NMR (125 MHz, CDCl_3_) δ 196.9, 169.0, 150.7, 136.5, 133.1, 120.4 (t, *J* = 271.1 Hz), 122.6, 120.4, 118.3, 58.6, 54.2, 39.7. IR (KBr): 3032, 2966, 1759, 1720, 1603, 1464, 1433, 1248, 1190, 1068, 1030 cm^−1^. HRMS (ESI) *m*/*z* Calcd: 295.0216 for C_12_H_10_O_3_F_2_SNa (M + Na)^+^, Found 295.0228.

*Methyl 2-((difluoromethyl)thio)-6-methoxy-1-oxo-2,3-dihydro-1H-indene-2-carboxylate* (**3c**) [24,26]. Yellow oil (31.1 mg, 51%, 90% ee). [α]^25^_D_ = −15.7 (*c* = 0.64, CHCl_3_). The ee value was determined by HPLC analysis using a Chiralpack IB3 column (hexane/*i*PrOH = 95:5, flow rate: 0.5 mL/min, t_R_ (minor) = 23.9 min (integral = 5.1%), t_R_ (major) = 25.9 min (integral = 94.9%). ^1^H-NMR (300 MHz, CDCl_3_) δ 7.52 (t, *J* = 55.7 Hz, 1H), 7.25–7.38 (m, 3H), 3.95 (d, *J* = 17.6 Hz, 1H), 3.86 (s, 3H), 3.82 (s, 3H), 3.20 (d, *J* = 17.6 Hz, 1H). ^19^F-NMR (282 MHz, CDCl_3_) δ −92.1 (dd, *J* = 251.7, 55.2 Hz, 1F), −93.5 (dd, *J* = 250.9, 56.0 Hz, 1F). HRMS (ESI) *m*/*z* Calcd: 325.0322 for C_13_H_12_O_4_F_2_NaS (M + Na)^+^ Found: 325.0325.

*Methyl 2-((difluoromethyl)thio)-5,6-dimethoxy-1-oxo-2,3-dihydro-1H-indene-2-carboxylate* (**3d**) [26]. Brown solid (36.2 mg, 54%, 94% ee). [α]^25^_D_ = −12.3 (*c* = 0.70, CHCl_3_). The ee value was determined by HPLC analysis using a Chiralpack OD-3 column (hexane/*i*PrOH = 95:5, flow rate: 1.0 mL/min, t_R_ (minor) = 27.2 min (integral = 3.2%), t_R_ (major) = 29.3 min (integral = 96.8%). ^1^H-NMR (300 MHz, CDCl_3_) δ 7.52 (t, *J* = 55.7 Hz, 1H), 7.27 (s, 1H), 6.88 (s, 1H), 4.00 (s, 3H), 3.93 (m, 4H), 3.82 (s, 3H), 3.21 (d, *J* = 17.6 Hz, 1H). ^19^F-NMR (282 MHz, CDCl_3_) δ −91.9 (dd, *J* = 250.9, 56.0 Hz, 1F), −93.5 (dd, *J* = 250.9, 56.0 Hz, 1F). HRMS (ESI) *m*/*z* Calcd: 355.0428 for C_14_H_14_O_5_F_2_SNa (M + Na)^+^ Found: 355.0427.

*Methyl 5-bromo-2-((difluoromethyl)thio)-1-oxo-2,3-dihydro-1H-indene-2-carboxylate* (**3e**). Brown solid (22.6 mg, 32%, 88% ee). M.p. 58.2–63.8 °C. [α]^25^_D_ = −2.5 (*c* = 1.1, CHCl_3_). The ee value was determined by HPLC analysis using a Chiralpack OD-3 column (hexane/*i*PrOH = 95:5, flow rate: 1.0 mL/min, t_R_ (minor) = 10.6 min (integral = 5.9%), t_R_ (major) = 12.0 min (integral = 94.0%). ^1^H-NMR (300 MHz, CDCl_3_) δ 7.72–7.63 (m, 3H), 7.45 (t, *J* = 53.3 Hz, 1H), 4.03 (d, *J* = 18.5 Hz, 1H), 3.83 (s, 3H), 3.27 (d, *J* = 17.9 Hz, 1H). ^19^F-NMR (282 MHz, CDCl_3_) δ −91.9 (dd, *J* = 250.9, 56.0 Hz, 1F), −93.5 (dd, *J* = 250.0, 55.2 Hz, 1F). ^13^C-NMR (125 MHz, CDCl_3_) δ 195.7, 168.5, 152.2, 132.5, 132.2, 132.0, 129.8, 127.1, 120.3 (t, *J* = 271.6 Hz), 58.768, 54.4, 39.3. IR (KBr): 2958, 1747, 1709, 1591, 1425, 1317, 1259, 1209, 1057, 1030 cm^−1^. HRMS (ESI) *m*/*z* Calcd: 372.9322 for C_12_H_9_O_3_F_2_NaSBr (M + Na)^+^ Found: 372.9309.

*Methyl 2-((difluoromethyl)thio)-5-fluoro-1-oxo-2,3-dihydro-1H-indene-2-carboxylate* (**3f**). Yellow oil (36.5 mg, 63%, 86% ee). [α]^25^_D_ = + 6.5 (*c* = 0.77, CHCl_3_). The ee value was determined by HPLC analysis using a Chiralpack OD-3 column (hexane/*i*PrOH = 95:5, flow rate: 1.0 mL/min, t_R_ (minor) = 11.8 min (integral = 6.8%), t_R_ (major) = 13.3 min (integral = 93.2%). ^1^H-NMR (300 MHz, CDCl_3_) δ 7.86 (dd, *J* = 8.2, 5.3 Hz, 1H), 7.48 (t, *J* = 55.5 Hz, 1H), 7.15–7.21 (m, 2H), 4.05 (d, *J* = 17.9 Hz, 1H), 3.82 (s, 3H), 3.28 (d, *J* = 17.9 Hz, 1H). ^19^F-NMR (282 MHz, CDCl_3_) δ −91.9 (dd, *J* = 250.9, 56.0 Hz, 1F), −93.5 (dd, *J* = 250.0, 55.2 Hz, 1F), −99.6 (dd, *J* = 13.8 Hz, 1F). ^13^C-NMR (125 MHz, CDCl_3_) δ 194.9, 168.4, 168.0 (d, *J* = 259.8 Hz), 153.6 (d, *J* = 10.9 Hz), 128.3 (d, *J* = 10.9 Hz), 117.2 (d, *J* = 23.6 Hz), 113.2 (d, *J* = 22.7 Hz), 58.8, 54.2, 39.4. IR (neat): 3074, 2958, 1747, 1720, 1618, 1595, 1429, 1255, 1068, 1041 cm^−1^. HRMS (ESI) *m*/*z* Calcd: 313.0122 for C_12_H_9_O_3_F_3_NaS (M + Na)^+^ Found: 313.0120.

*Ethyl 2-((difluoromethyl)thio)-1-oxo-2,3-dihydro-1H-indene-2-carboxylate* (**3g**). Yellow oil (35 mg, 61%, 85% ee). [α]^25^_D_ = + 3.8 (*c* = 0.46, CHCl_3_). The ee value was determined by HPLC analysis using a Chiralpack IB3 column (hexane/*i*PrOH = 99:1, flow rate: 1.0 mL/min, t_R_ (minor) = 24.5 min (integral = 7.6%), t_R_ (major) = 27.3 min (integral = 92.4%). ^1^H-NMR (300 MHz, CDCl_3_) δ 7.85 (d, *J* = 8.2 Hz, 1H), 7.70 (d, *J* = 15.9 Hz, 1H), 7.53 (t, *J* = 51.2 Hz, 1H), 7.45–7.50 (m, 2H), 4.29 (q, *J* = 7.2 Hz, 2H), 4.04 (d, *J* = 17.9 Hz, 1H), 3.25 (d, *J* = 17.9 Hz, 1H), 1.29 (t, *J* = 7.2 Hz, 3H). ^19^F-NMR (282 MHz, CDCl_3_) δ −91.9 (dd, *J* = 250.9, 56.0 Hz, 1F), −93.3 (dd, *J* = 250.0, 56.9 Hz, 1F). ^13^C-NMR (125 MHz, CDCl_3_) δ 197.1, 168.4, 150.7, 136.5, 133.2, 128.8, 126.4, 126.0, 120.5 (t, *J* = 270.7 Hz), 63.6, 58.5, 39.6, 14.1. IR (neat): 2985, 1739, 1720, 1606, 1468, 1271, 1244, 1213, 1182, 1065, 1034 cm^−1^. HRMS (ESI) *m*/*z* Calcd: 309.0373 for C_13_H_12_O_3_F_2_NaS (M + Na)^+^ Found: 309.0351.

*Methyl 2-((difluoromethyl)thio)-2-Methyl-3-Oxo-3-Phenylpropanoate* (**3h**). Colorless oil (19.5 mg, 36%, 12% ee). The ee value was determined by HPLC analysis using a Chiralpack OD-3 column (hexane/*i*PrOH = 99:1, flow rate: 0.53 mL/min, t_R_ (minor) = 40.2 min (integral = 43.8%), t_R_ (major) = 45.3 min (integral = 52.5%). ^1^H-NMR (300 MHz, CDCl_3_) δ 7.91 (d, *J* = 7.4 Hz, 2H), 7.56–7.61 (m, 1H), 7.43–7.48 (m, 2H), 6.89 (t, *J* = 55.9 Hz, 1H), 3.73 (s, 3H), 1.97 (s, 3H). ^19^F-NMR (282 MHz, CDCl_3_) δ −92.7 (d, *J* = 55.2 Hz, 2F). HRMS (ESI) *m*/*z*: 297 (M + Na)^+^.

## 4. Conclusions

In summary, we have described the first asymmetric electrophilic difluoromethylthiolation of β-keto esters by means of a difluoromethanesulfonyl hypervalent iodonium ylide. The traceless use of chiral amines as chiral auxiliary allowed the synthesis of enantioenriched indanone-based α-SCF_2_H β-keto esters in up to 93% ee value. We believe that this synthetic approach to enantiomerically enriched indanone scaffolds will create interest for the design of new biologically attractive drug candidates having α-SCF_2_H indanone moiety. While tetralone-type and acyclic substrates failed to react efficiently, the improvement of the results could be theoretically possible by using chiral amines with electron withdrawing groups. This investigation is ongoing in our laboratory.

## Figures and Tables

**Table 1:**
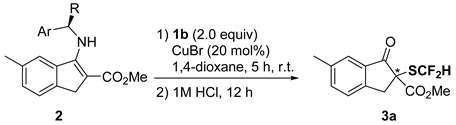
Screening of chiral amines.

Entry	Ar	R	2	Yield (%) ^1^	Ee (%) ^2^
1	Ph	Me	**2a**	79	57
2	Ph	Et	**2b**	79	46
3	1-Naphthyl	Me	**2c**	57	62
4	4-MeC_6_H_4_	Me	**2d**	80	57
5	4-MeOC_6_H_4_	Me	**2e**	78	49

^1^ Yields for isolated pure products. ^2^ Ee values were determined by HPLC analysis [35]. The asterisk indicates the chiral center in the molecule **3a**.

**Table 2:**
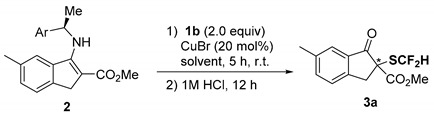
Optimization of the reaction conditions.

Entry	Ar	2	Solvent	Yield (%) ^1^	Ee (%) ^2^
1	Ph	**2a**	1,4-dioxane	79	57
2	Ph	**2a**	THF	75	56
3	Ph	**2a**	CH_2_Cl_2_	74	67
4	Ph	**2a**	CHCl_3_	75	68
5	Ph	**2a**	Toluene	76	69
6	1-Naphthyl	**2c**	1,4-dioxane	57	62
7	1-Naphthyl	**2c**	CH_2_Cl_2_	52	88
8	1-Naphthyl	**2c**	CHCl_3_	65	87
9	1-Naphthyl	**2c**	Toluene	59	88
10	1-Naphthyl	**2c**	CH_3_CN	0	–

^1^ Yields for isolated pure products. ^2^ Ee values were determined by HPLC analysis [35]. The asterisk indicates the chiral center in the molecule **3a**.

## Supplementary Materials

The following are available online, ^1^H, ^13^C and ^19^F NMR spectra of compounds **3** and HPLC data of compounds **3**.
