# Supplementary material for: Asymmetric Electrophilic Difluoromethylthiolation of Indanone-Based β-Keto Esters Using Difluoromethanesulfonyl Hypervalent Iodonium Ylides
Source: Molecules. 2019 Jan 9;24(2):221. doi: 10.3390/molecules24020221 (PMC6359606; doi:10.3390/molecules24020221)
Supplement: Supplementary file 1 [file molecules-24-00221-s001.pdf]

Supplementary Materials

Asymmetric Electrophilic Difluoromethylthiolation of Indanone-Based β-Keto Esters using Difluoromethanesulfonyl Hypervalent Iodonium Ylides

Satoshi Gondo,[1] Okiya Matsubara,[1] Hélène Chachignon,[2] Yuji Sumii,[1] Dominique Cahard,[2] and Norio Shibata[1,3,*]

1 *Department of Nanopharmaceutical Sciences, Nagoya Institute of Technology, Gokiso, Showa-ku, Nagoya 466-8555, Japan; cjh11141@nitech.jp (O. M.); 29411074@stn.nitech.ac.jp (S. G.); sumii.yuji@nitech.ac.jp (Y . S.) Should be Sumii's e-mail*

2 *CNRS, UMR 6014 COBRA, Normandie Université, 1 Rue Tesnière, F-76821 Mont-Saint-Aignan Cedex, France; helene.chachignon@insa-rouen.fr. (H. C.); dominique.cahard@univ-rouen.fr. (D. C.)*

3 *Institute of Advanced Fluorine-Containing Materials, Zhejiang Normal University, 688 Yingbin Avenue, 321004 Jinhua, China*

* *Correspondence: nozshiba@nitech.ac.jp; Tel./Fax: +81-52-735-7543*

Table of contents

^{1}H, ^{13}C and ^{19}F NMR spectra of compound 3 2

HPLC data of compound 3 12

^{1}H, ^{13}C and ^{19}F NMR spectra of compound 3

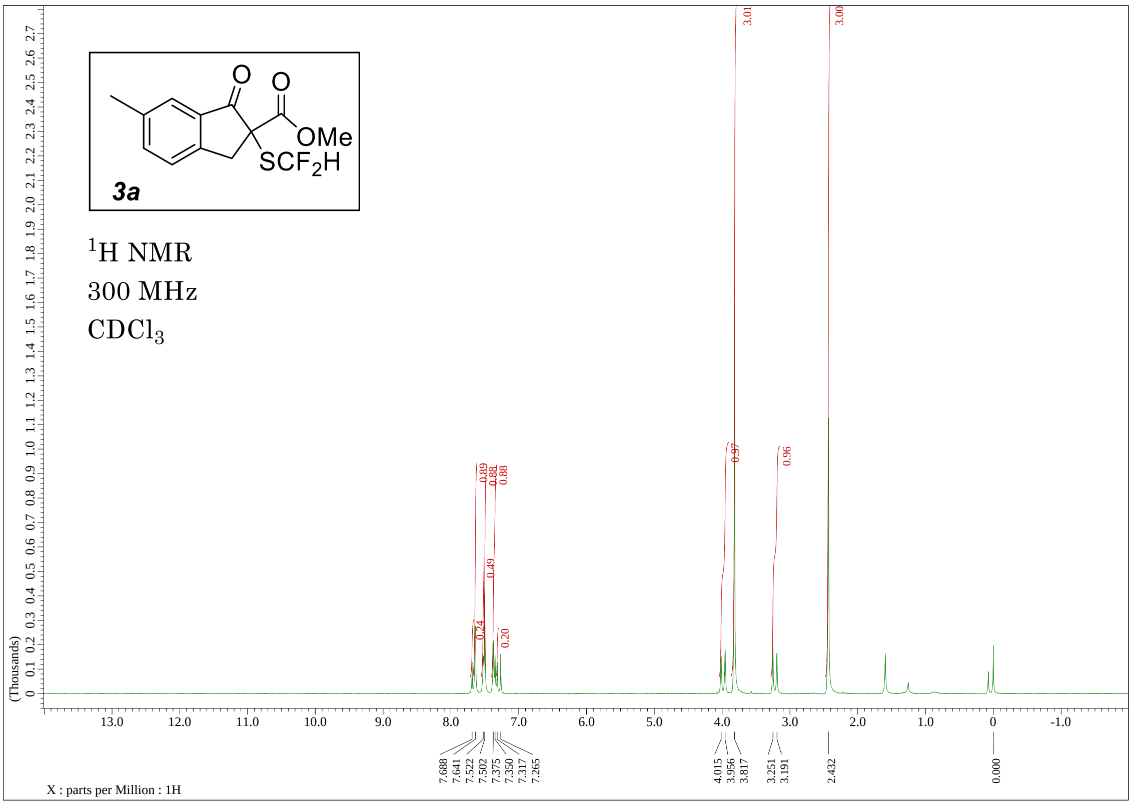

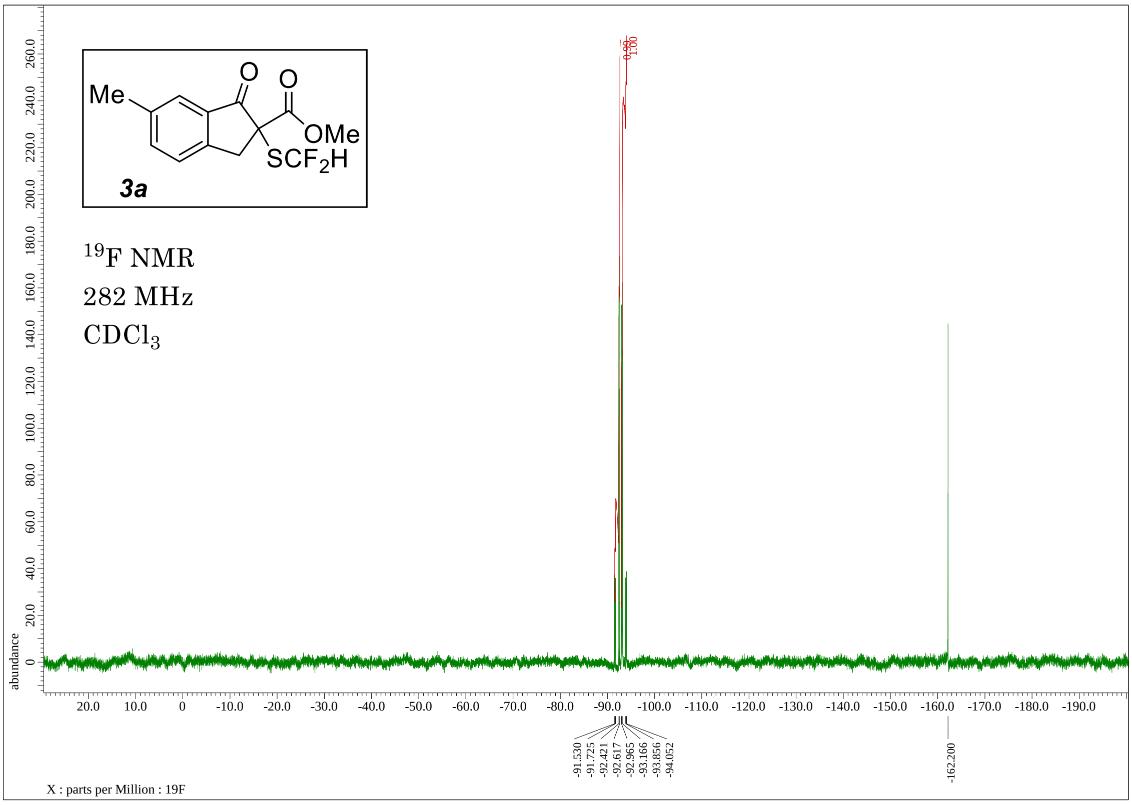

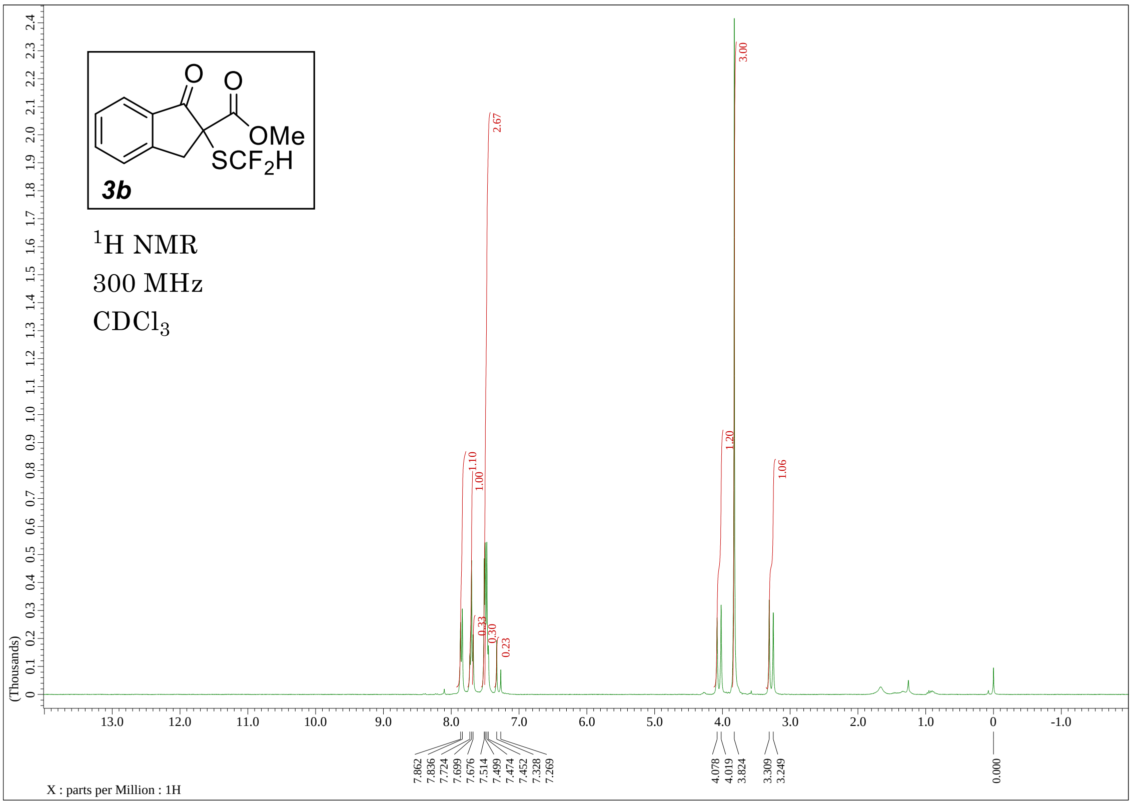
3b
^{1}H NMR
300 MHz
$CDCl_3$
(Thousands)
X : parts per Million : 1H
7.862 7.836 7.724 7.699 7.676 7.514 7.499 7.474 7.452 7.328 7.269
4.078 4.019 3.824 3.309 3.249
0.000
1.10 1.00 2.67 0.33 0.30 0.23 1.20 3.00 1.06

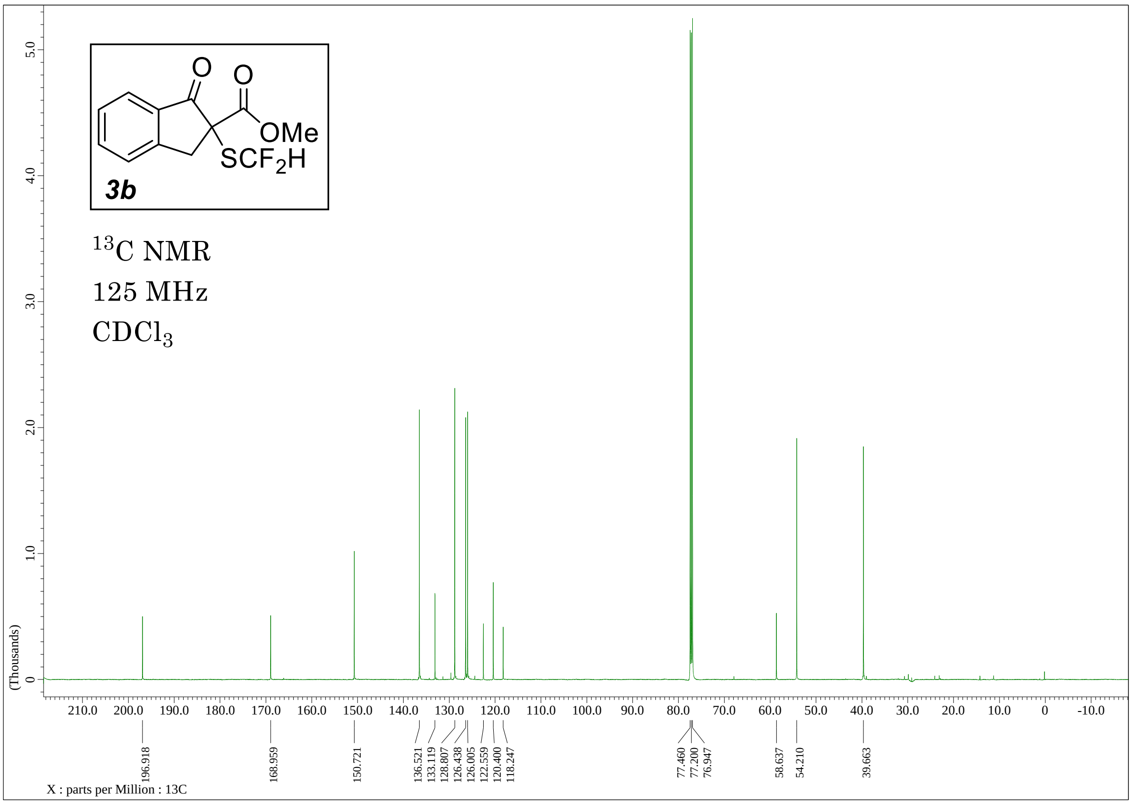
3b
^{13}C NMR
125 MHz
$CDCl_3$
(Thousands)
X : parts per Million : 13C
196.918
168.959
150.721
136.521 133.119 128.807 126.438 126.005 122.559 120.400 118.247
77.460 77.200 76.947
58.637 54.210 39.663

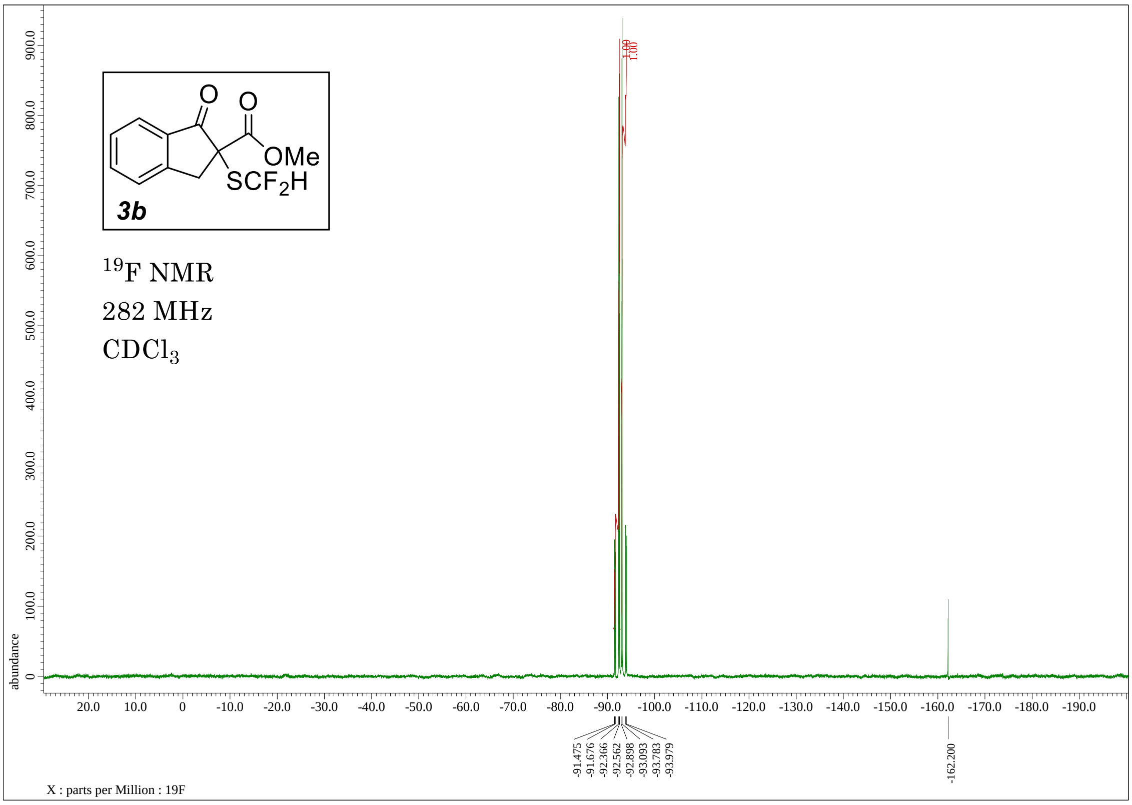
3b
^{19}F NMR
282 MHz
$CDCl_3$
abundance
X : parts per Million : 19F
-91.475
-91.676
-92.366
-92.562
-92.898
-93.093
-93.783
-93.979
-162.200

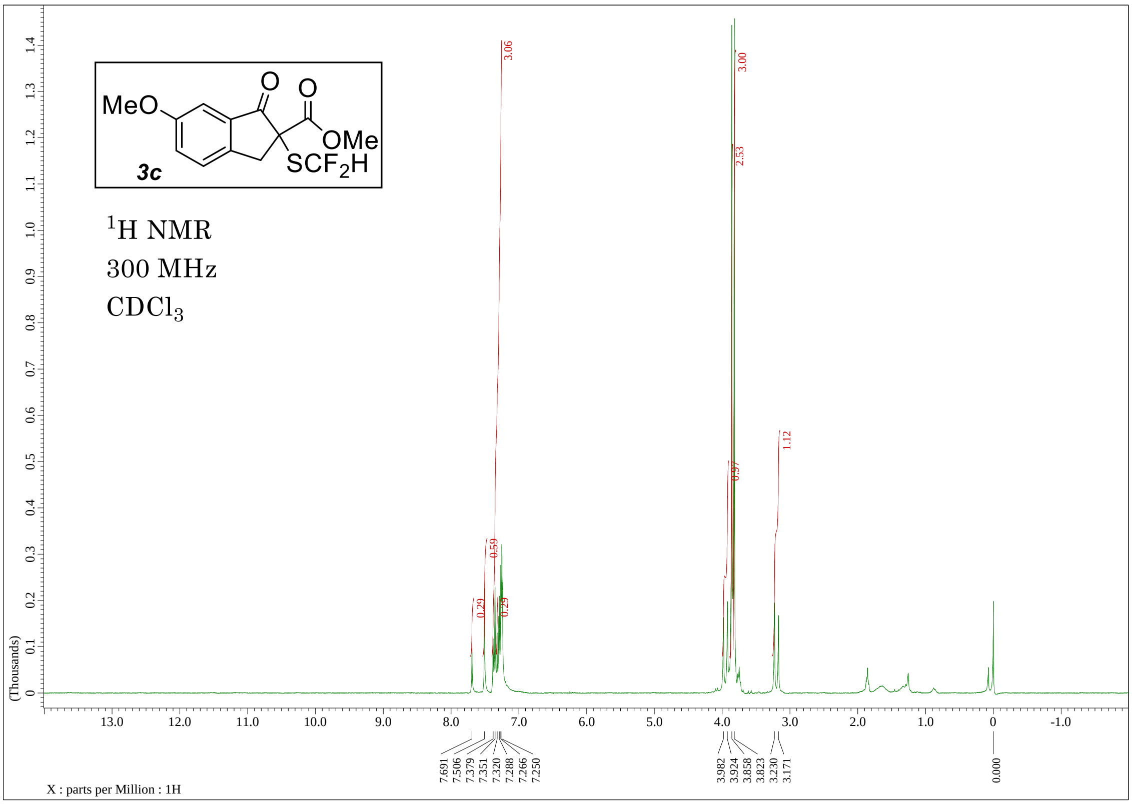
MeO
OMe
SCF_2H
3c
^{1}H NMR
300 MHz
$CDCl_3$
(Thousands)
X : parts per Million : 1H
7.691
7.506
7.379
7.351
7.320
7.288
7.266
7.250
3.982
3.924
3.858
3.823
3.230
3.171
0.000

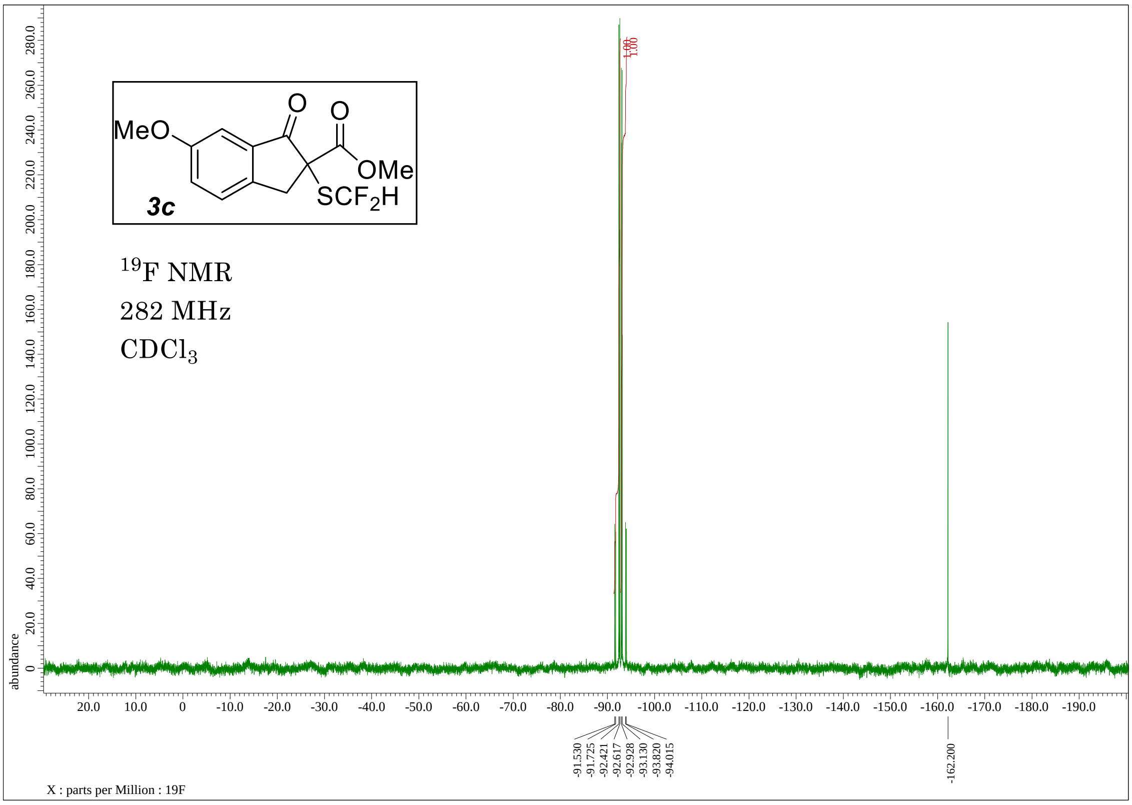
MeO
O
O
OMe
SCF2H
3c
19F NMR
282 MHz
CDCl3
abundance
X : parts per Million : 19F
-91.530
-91.725
-92.421
-92.617
-92.928
-93.130
-93.820
-94.015
-162.200

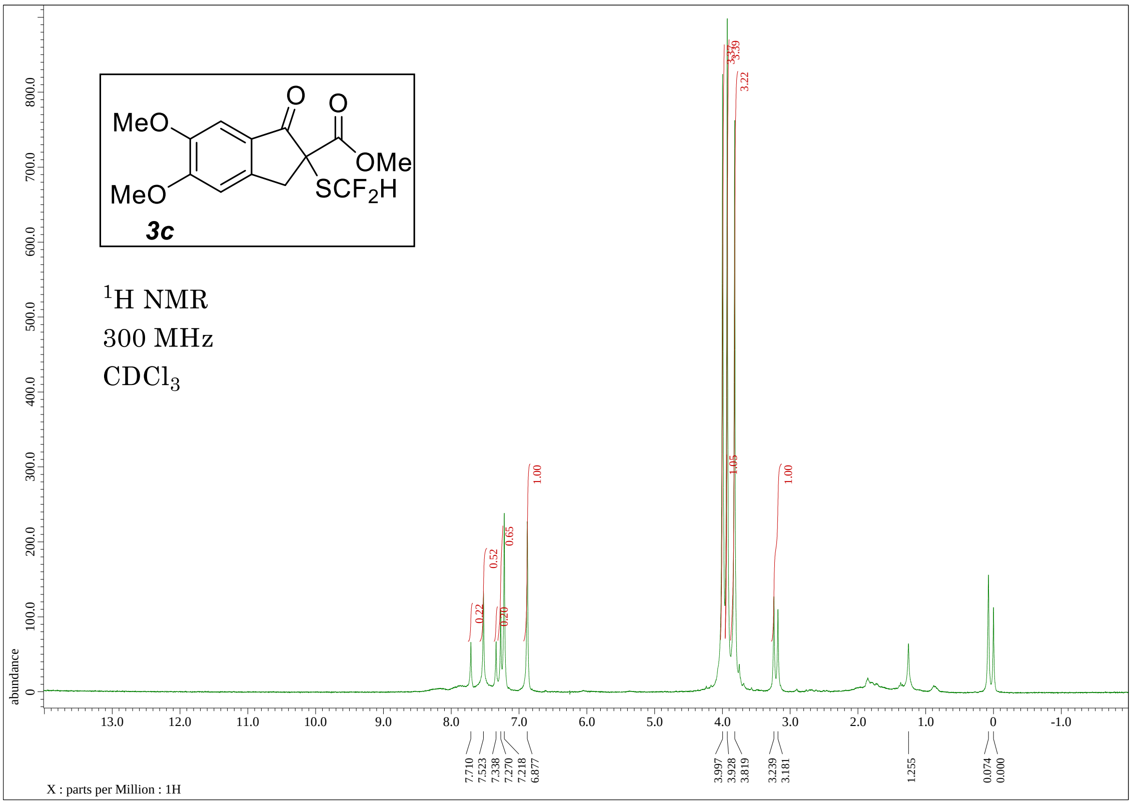
MeO
MeO
O
O
OMe
SCF2H
3c
1H NMR
300 MHz
CDCl3
abundance
X : parts per Million : 1H
7.710
7.523
7.338
7.270
7.218
6.877
3.997
3.928
3.819
3.239
3.181
1.255
0.074
0.000

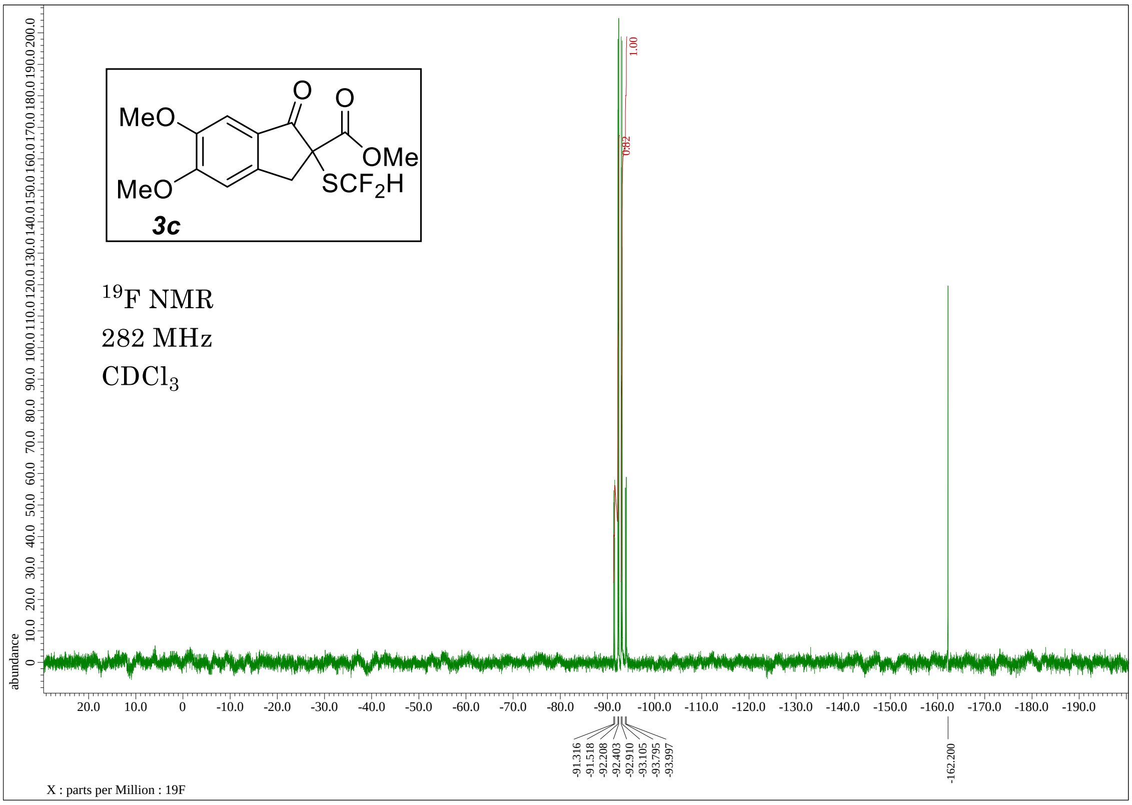
MeO
MeO
O
O
OMe
SCF2H
3c
19F NMR
282 MHz
CDCl3
abundance
X : parts per Million : 19F
-91.316
-91.518
-92.208
-92.403
-92.910
-93.105
-93.795
-93.997
-162.200
1.00
0.82

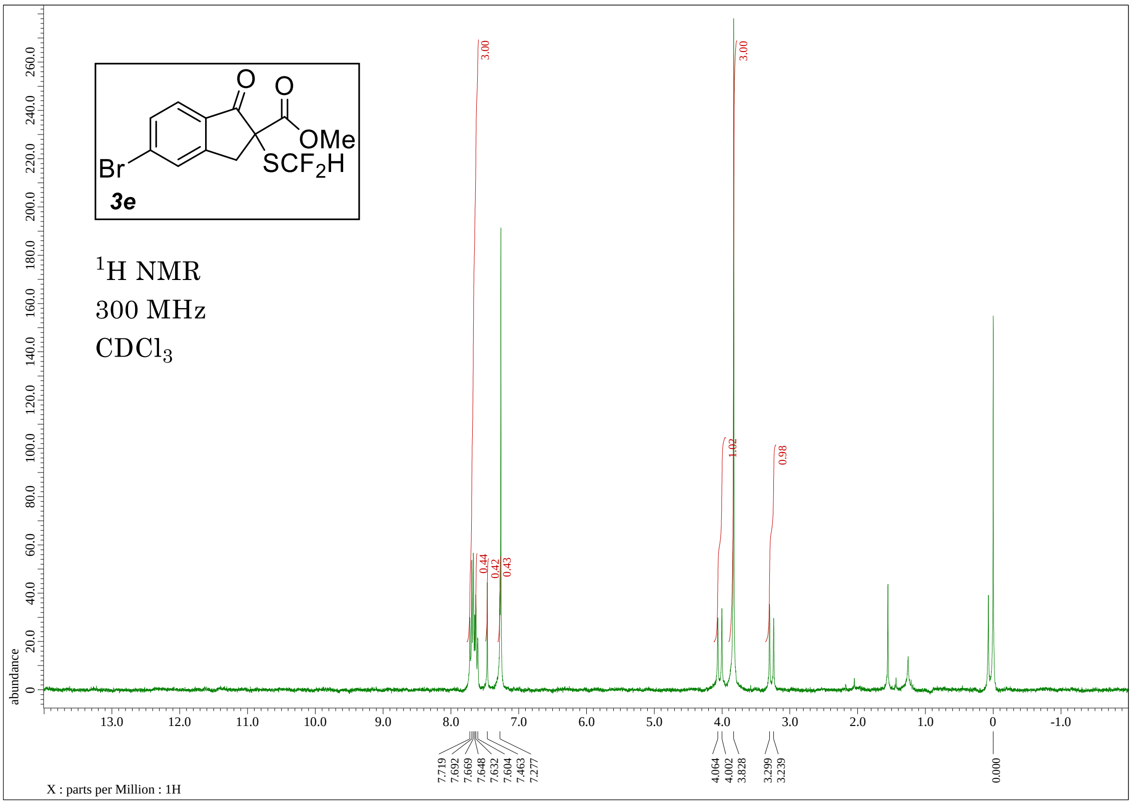
Br
O
O
OMe
SCF2H
3e
1H NMR
300 MHz
CDCl3
abundance
X : parts per Million : 1H
7.719
7.692
7.669
7.648
7.632
7.604
7.463
7.277
4.064
4.002
3.828
3.299
3.239
0.000
3.00
0.44
0.42
0.43
1.02
3.00
0.98

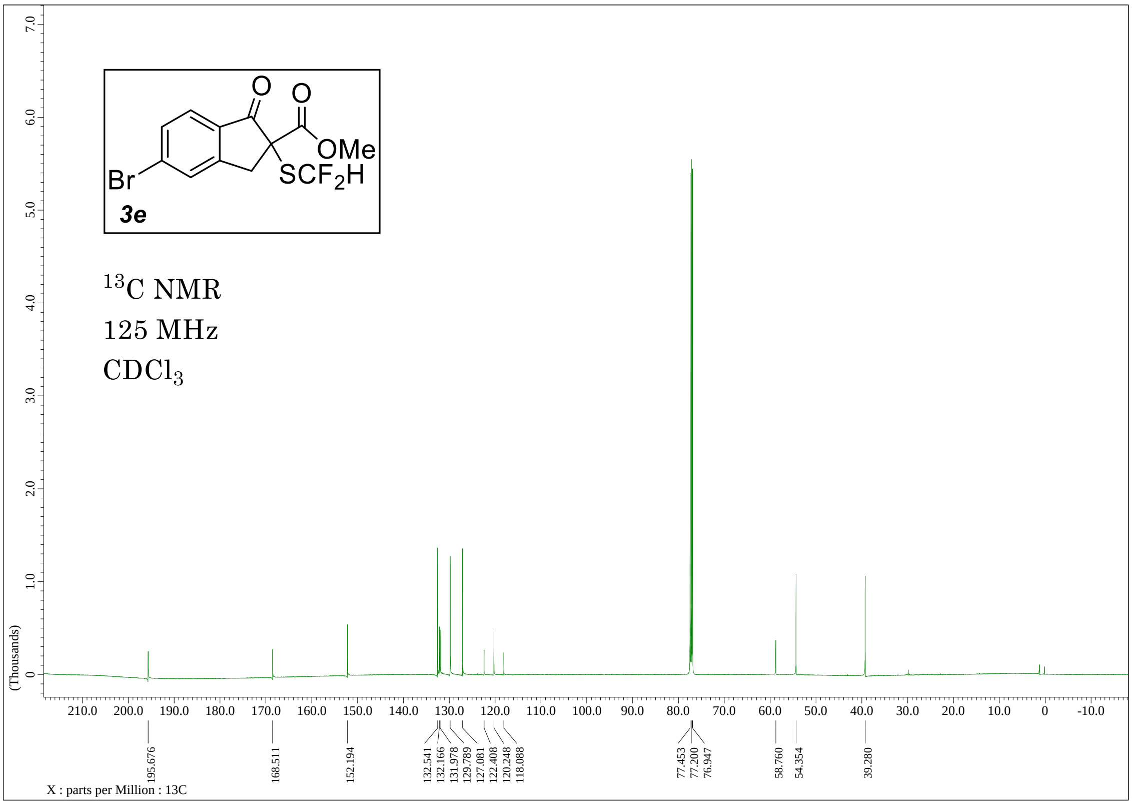

O
O
OMe
SCF2H
Br
3e
13C NMR
125 MHz
CDCl3
(Thousands)
X : parts per Million : 13C
195.676
168.511
152.194
132.541
132.166
131.978
129.789
127.081
122.408
120.248
118.088
77.453
77.200
76.947
58.760
54.354
39.280

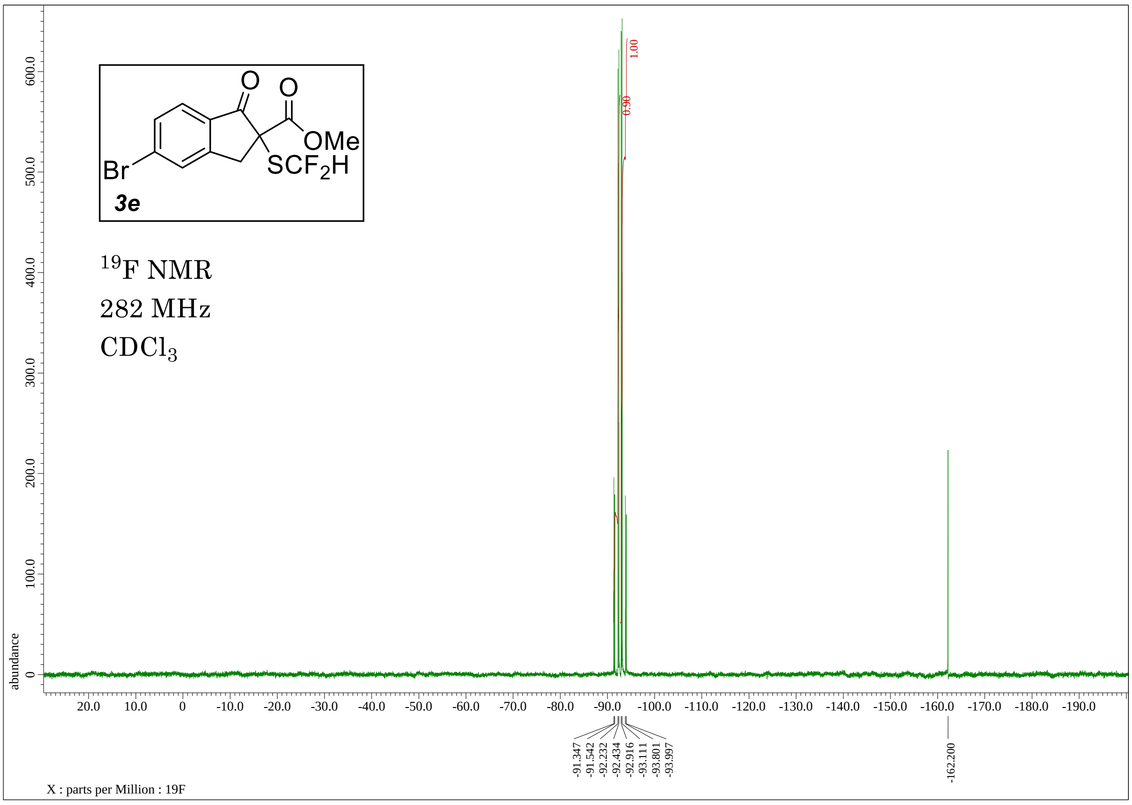

O
O
OMe
SCF2H
Br
3e
19F NMR
282 MHz
CDCl3
abundance
1.00
0.90
X : parts per Million : 19F
-91.347
-91.542
-92.232
-92.434
-92.916
-93.111
-93.801
-93.997
-162.200

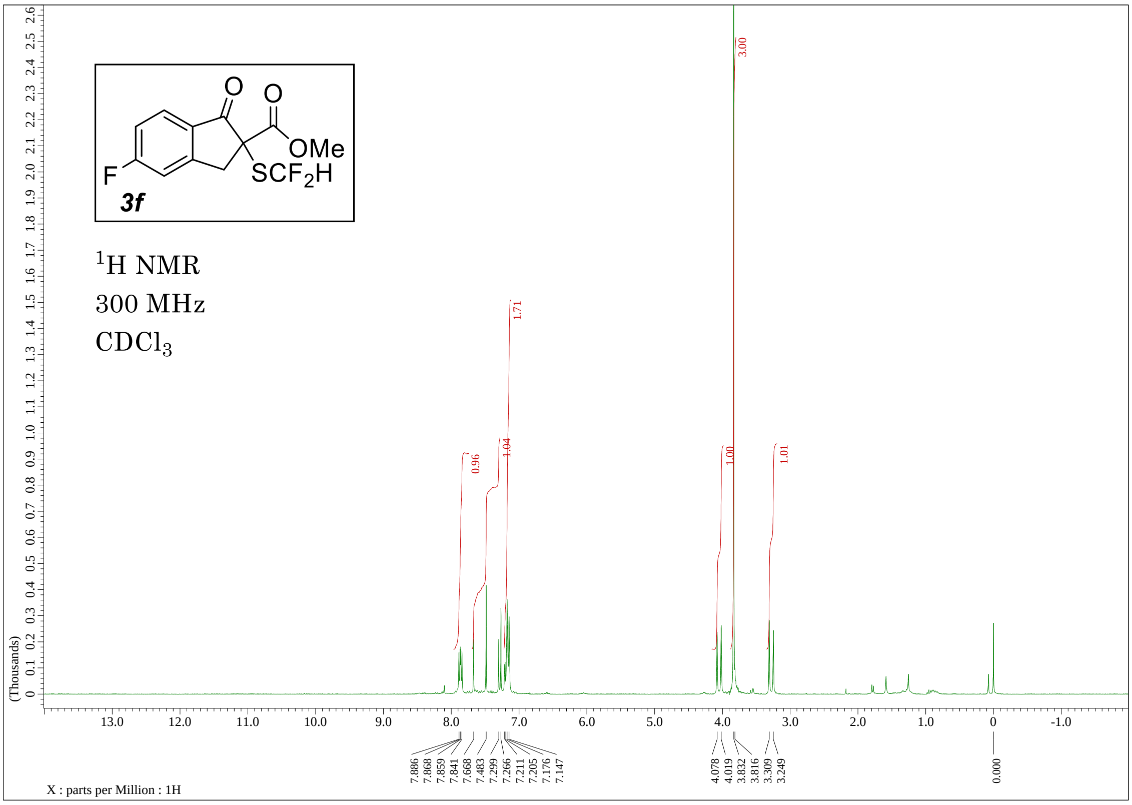
O
O
OMe
SCF2H
F
3f
1H NMR
300 MHz
CDCl3
3.00
1.71
0.96
1.04
1.00
1.01
(Thousands)
7.886
7.868
7.859
7.841
7.668
7.483
7.299
7.266
7.211
7.205
7.176
7.147
4.078
4.019
3.832
3.816
3.309
3.249
0.000
X : parts per Million : 1H

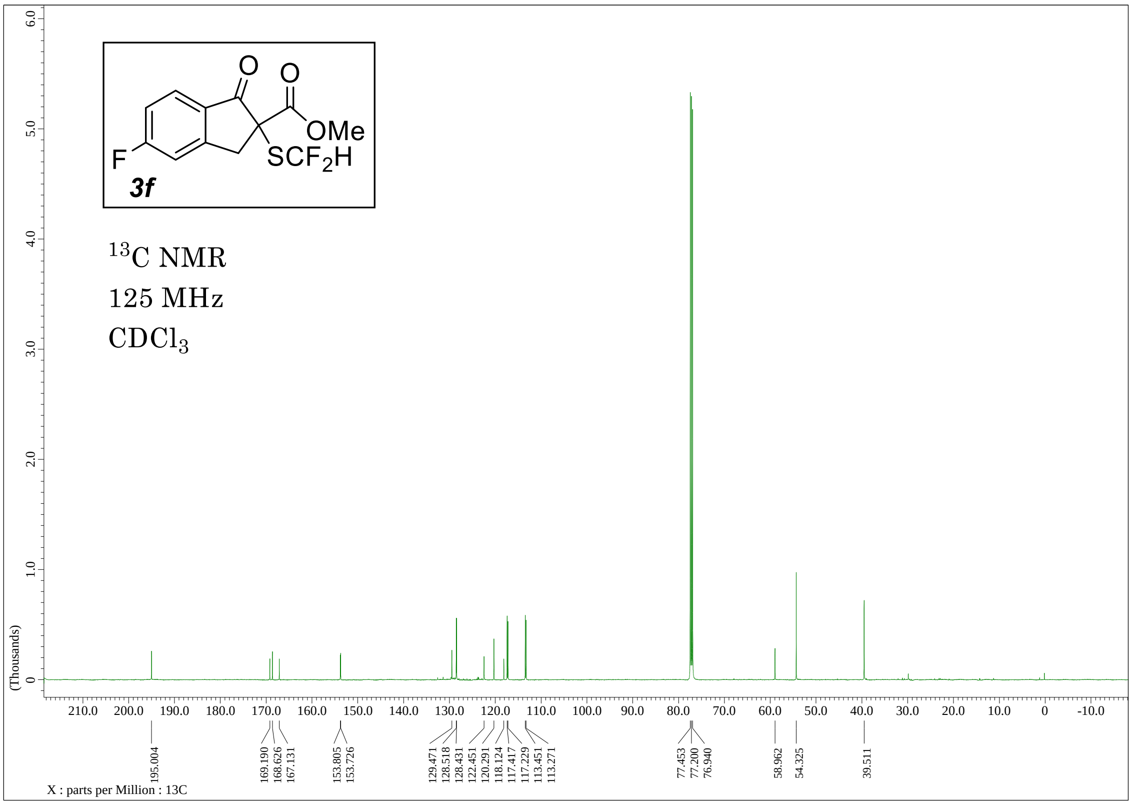
O
O
OMe
SCF2H
F
3f
13C NMR
125 MHz
CDCl3
(Thousands)
195.004
169.190
168.626
167.131
153.805
153.726
129.471
128.518
128.431
122.451
120.291
118.124
117.417
117.229
113.451
113.271
77.453
77.200
76.940
58.962
54.325
39.511
X : parts per Million : 13C

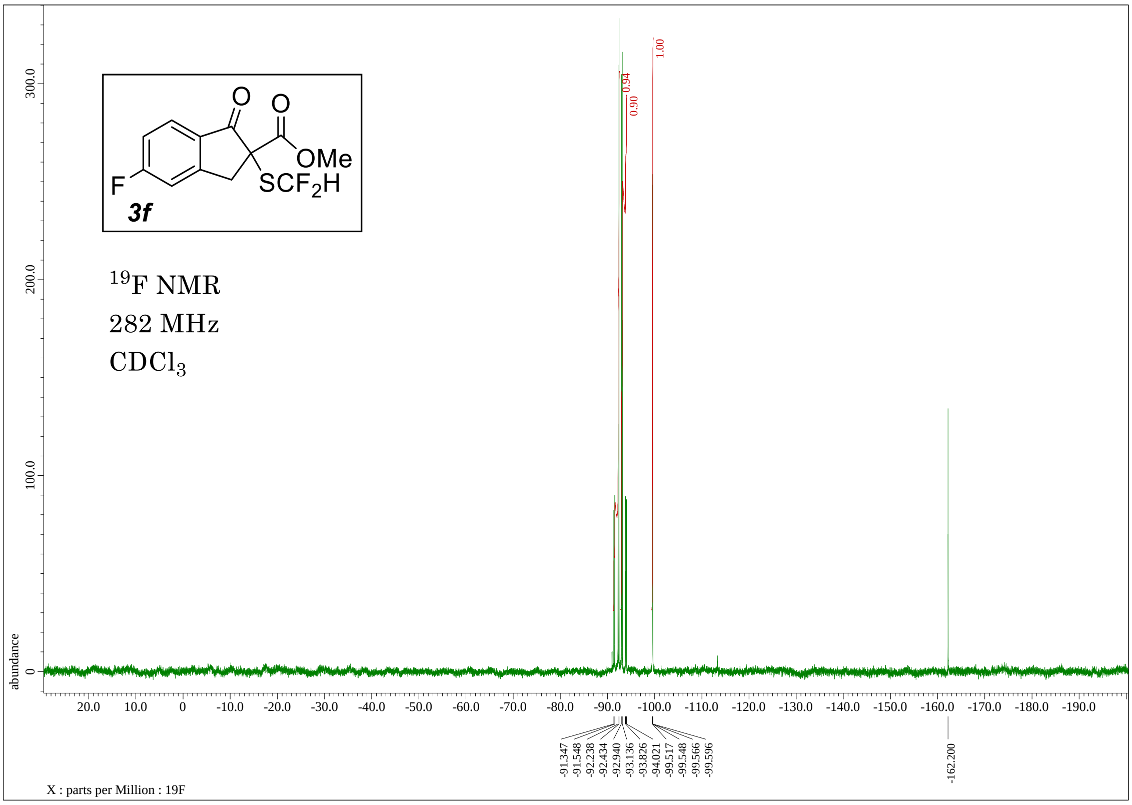

3f
OMe
SCF2H
F
19F NMR
282 MHz
CDCl3
abundance
X : parts per Million : 19F
-91.347
-91.548
-92.238
-92.434
-92.940
-93.136
-93.826
-94.021
-99.517
-99.548
-99.566
-99.596
-162.200

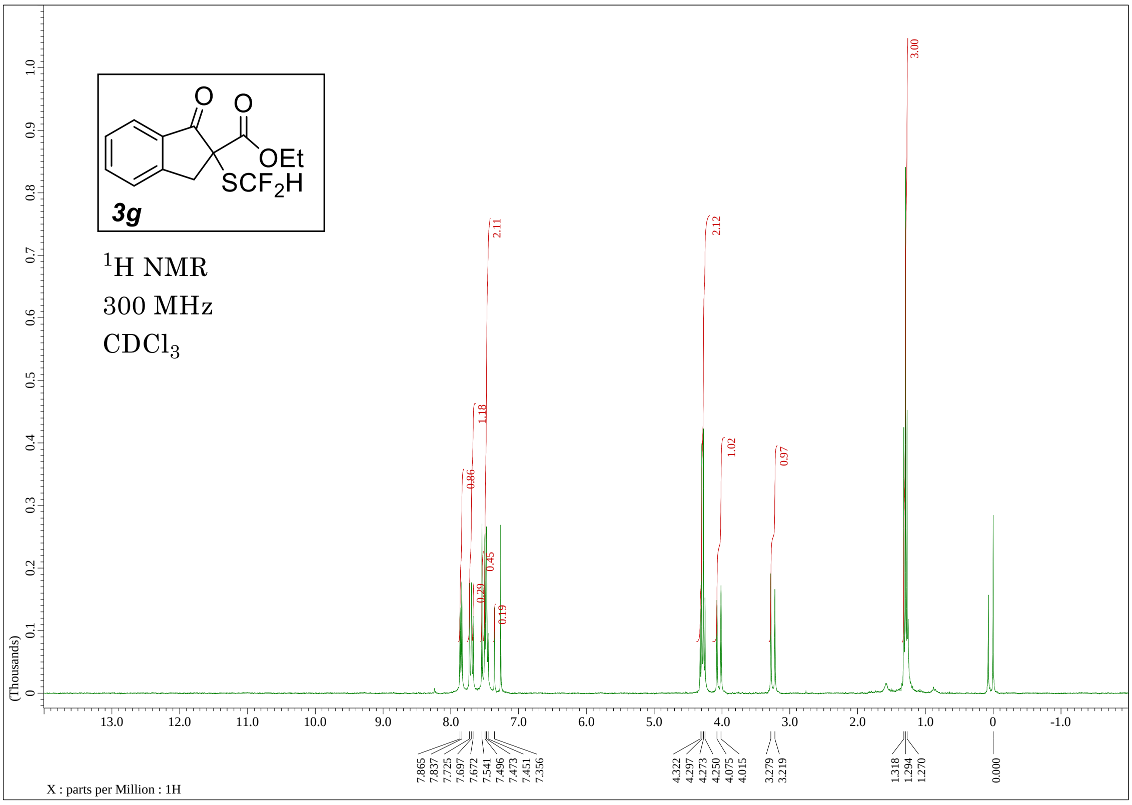

3g
OEt
SCF2H
1H NMR
300 MHz
CDCl3
(Thousands)
X : parts per Million : 1H
7.865
7.837
7.725
7.697
7.672
7.541
7.496
7.473
7.451
7.356
4.322
4.297
4.273
4.250
4.075
4.015
3.279
3.219
1.318
1.294
1.270
0.000

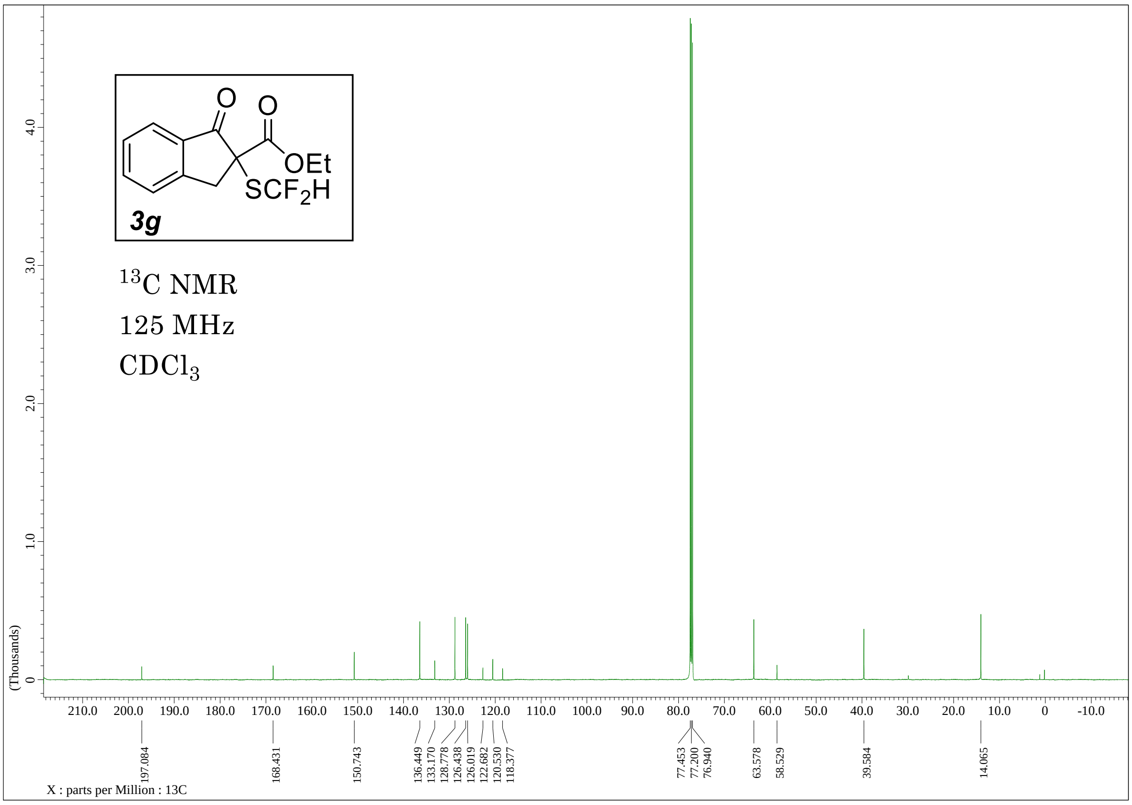
O
O
OEt
SCF2H
3g
13C NMR
125 MHz
CDCl3
(Thousands)
X : parts per Million : 13C
197.084
168.431
150.743
136.449
133.170
128.778
126.438
126.019
122.682
120.530
118.377
77.453
77.200
76.940
63.578
58.529
39.584
14.065

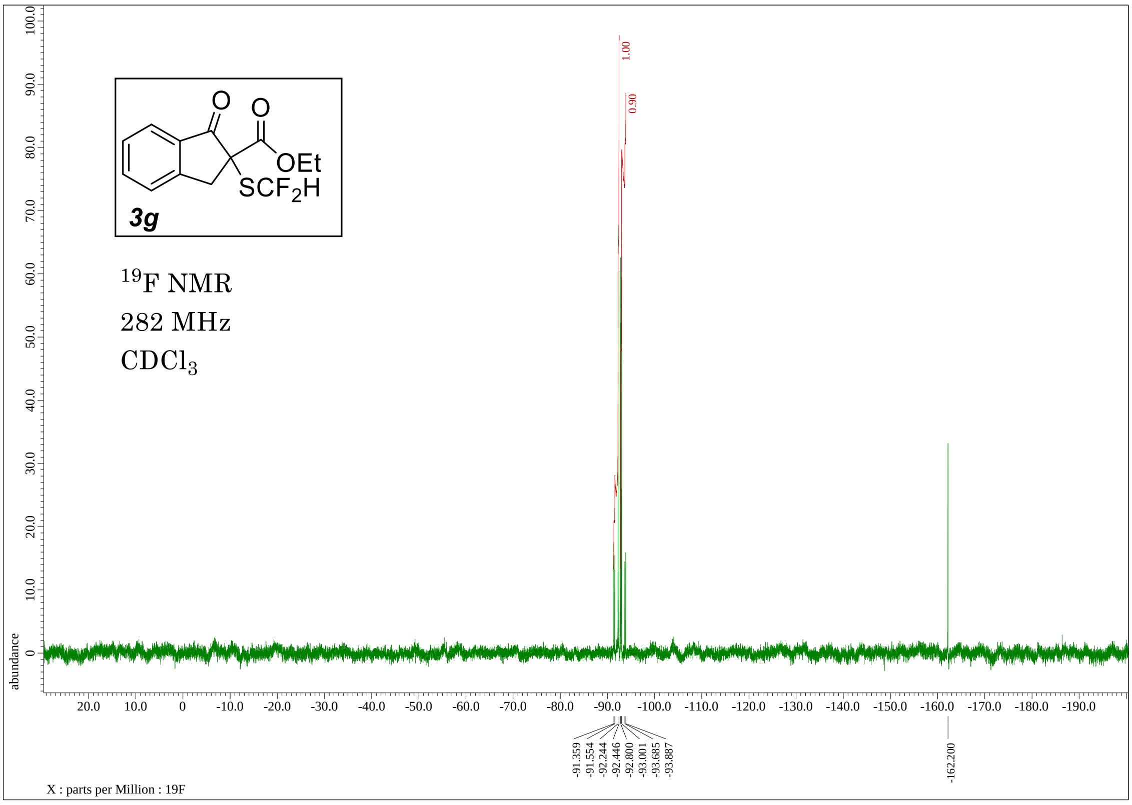
O
O
OEt
SCF2H
3g
19F NMR
282 MHz
CDCl3
abundance
1.00
0.90
X : parts per Million : 19F
-91.359
-91.554
-92.244
-92.446
-92.800
-93.001
-93.685
-93.887
-162.200

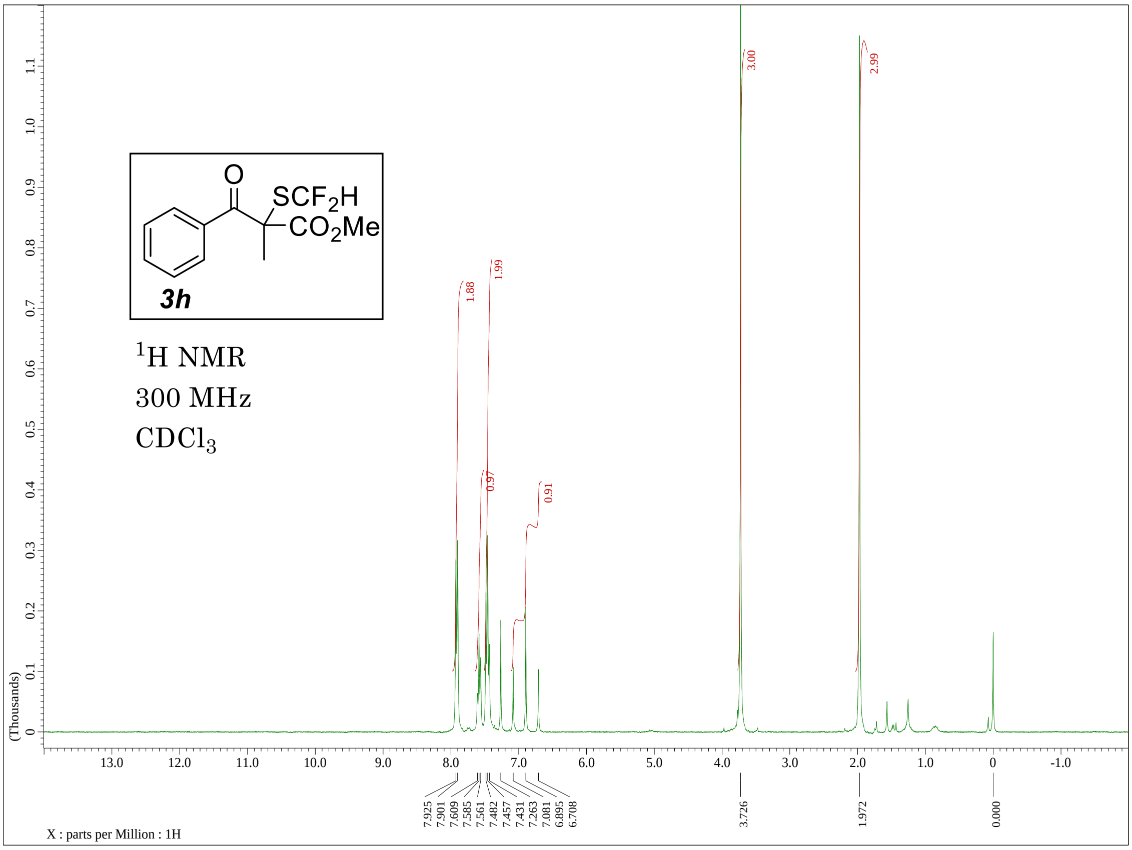
SCF2H
CO2Me
3h
1H NMR
300 MHz
CDCl3
X : parts per Million : 1H
(Thousands)

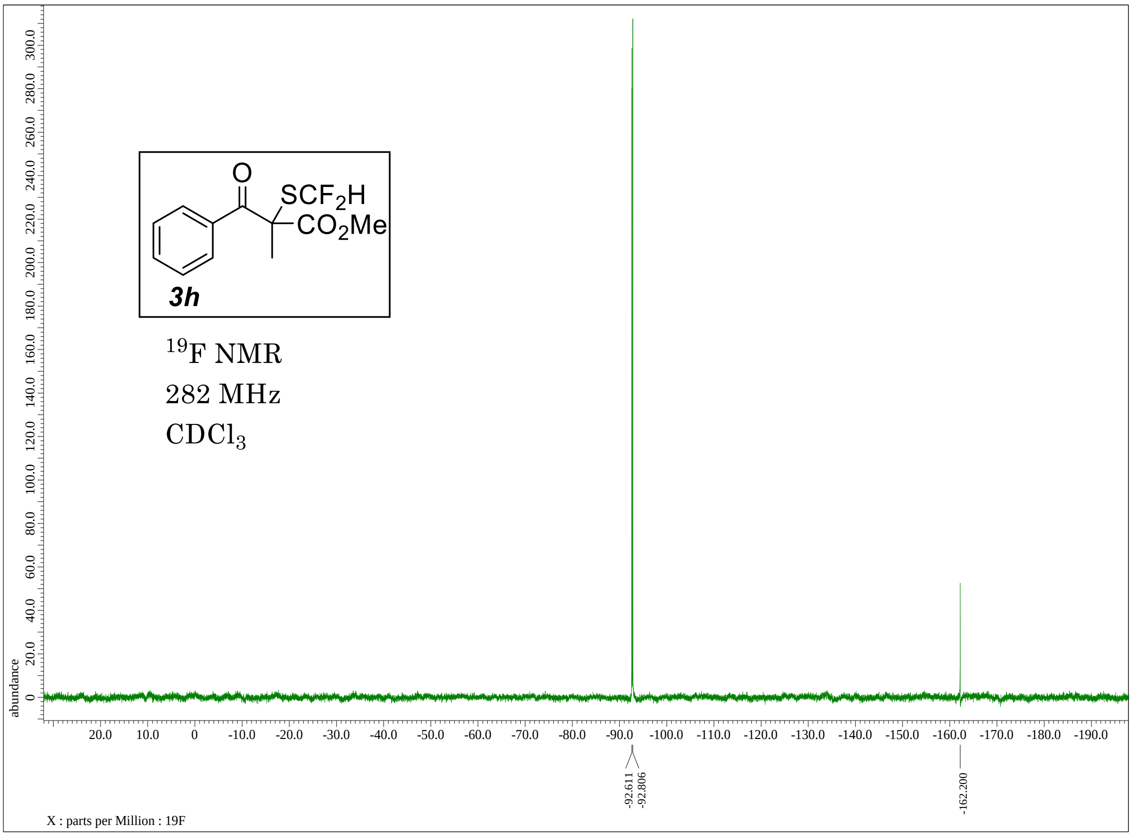
SCF2H
CO2Me
3h
19F NMR
282 MHz
CDCl3
X : parts per Million : 19F
abundance

HPLC data of compound 3

Methyl 2-((difluoromethyl)thio)-6-methyl-1-oxo-2,3-dihydro-1*H*-indene-2-carboxylate (3a)

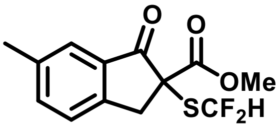

chiral pack IB3, Hexane/*i*PrOH = 98:2, 0.5 mL/min

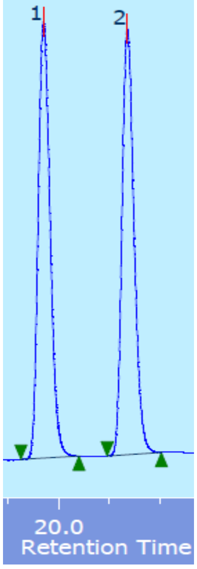

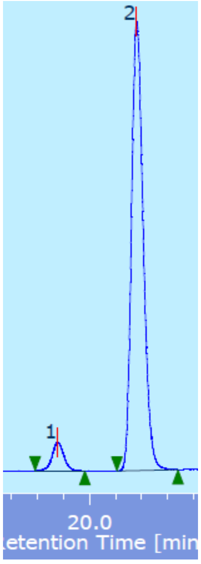

No.	tR (min)	Area (%)	High (%)
1	19.392	50.257	50.610
2	22.692	49.743	49.390

No.	tR (min)	Area (%)	High (%)
1	18.775	5.917	5.955
2	21.800	94.083	94.045

Methyl 2-((difluoromethyl)thio)-1-oxo-2,3-dihydro-1*H*-indene-2-carboxylate (3b)

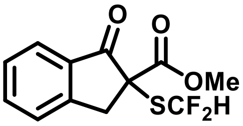

chiral pack IB3, Hexane/*i*PrOH = 98:2, 0.5 mL/min

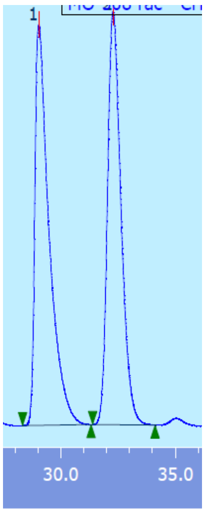

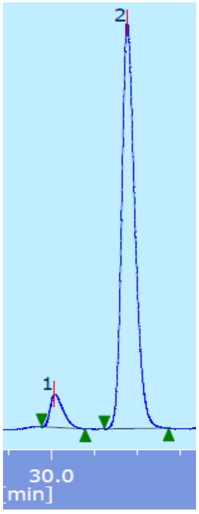

No.	tR (min)	Area (%)	High (%)
1	29.025	50.081	49.253
2	32.258	49.919	50.747

No.	tR (min)	Area (%)	High (%)
1	30.150	7.479	7.578
2	33.533	92.251	92.422

Methyl 2-((difluoromethyl)thio)-6-methoxy-1-oxo-2,3-dihydro-1*H*-indene-2-carboxylate (3c)

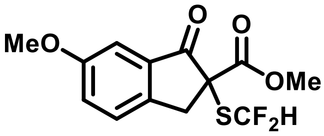

chiral pack IB3, Hexane/*i*PrOH = 95:5, 0.5 mL/min

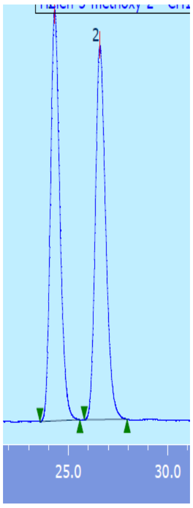

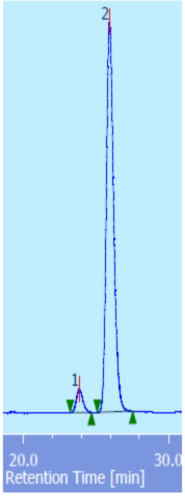

No.	tR (min)	Area (%)	High (%)
1	24.308	49.845	52.304
2	26.575	50.155	47.696

No.	tR (min)	Area (%)	High (%)
1	23.850	5.064	5.588
2	25.933	94.936	94.412

Methyl 2-((difluoromethyl)thio)-5,6-dimethoxy-1-oxo-2,3-dihydro-1*H*-indene-2-carboxylate (3d)

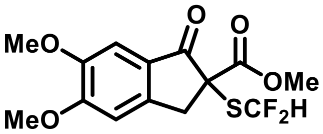

chiral pack OD-3, Hexane/*i*PrOH = 95:5, 1.0 mL/min

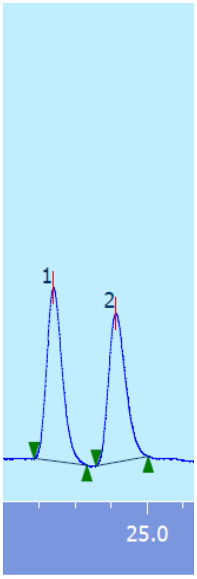

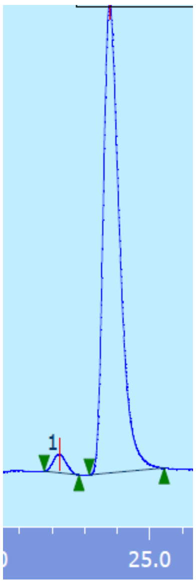

No.	tR (min)	Area (%)	High (%)
1	22.392	51.293	53.710
2	24.133	48.707	46.290

No.	tR (min)	Area (%)	High (%)
1	22.208	3.074	3.827
2	23.767	96.926	96.173

Methyl 5-bromo-2-((difluoromethyl)thio)-1-oxo-2,3-dihydro-1*H*-indene-2-carboxylate (3e)

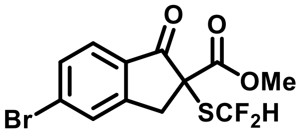

chiral pack OD-3, Hexane/*i*PrOH = 95:5, 1.0 mL/min

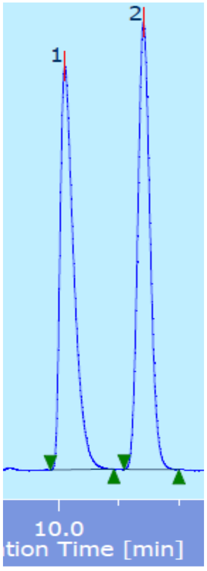

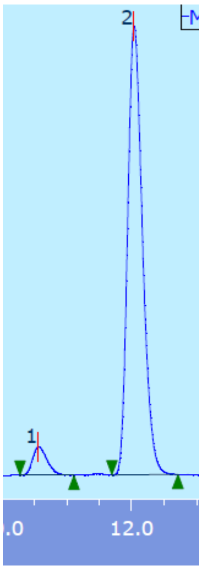

No.	tR (min)	Area (%)	High (%)
1	10.092	50.065	47.444
2	11.408	49.935	52.556

No.	tR (min)	Area (%)	High (%)
1	10.558	5.916	5.965
2	12.033	94.084	94.035

Methyl 2-((difluoromethyl)thio)-5-fluoro-1-oxo-2,3-dihydro-1*H*-indene-2-carboxylate (3f)

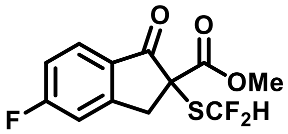

chiral pack OD-3, Hexane/*i*PrOH = 95:5, 1.0 mL/min

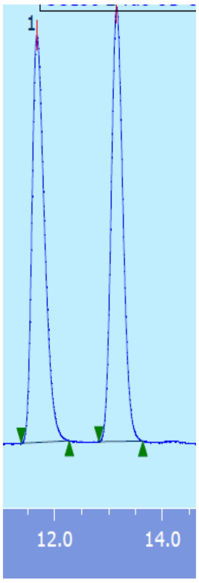

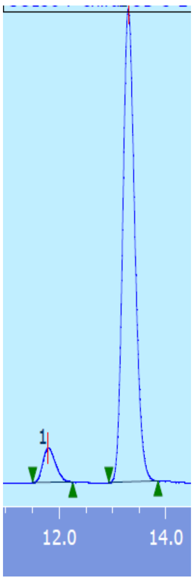

No.	tR (min)	Area (%)	High (%)
1	11.675	50.039	48.403
2	13.142	49.961	51.597

No.	tR (min)	Area (%)	High (%)
1	11.792	7.175	6.770
2	13.292	92.825	93.230

Ethyl 2-((difluoromethyl)thio)-1-oxo-2,3-dihydro-1*H*-indene-2-carboxylate (3g)

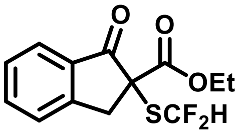

chiral pack IB3, Hexane/*i*PrOH = 99:1, 1.0 mL/min

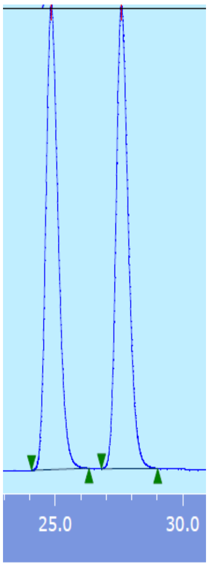

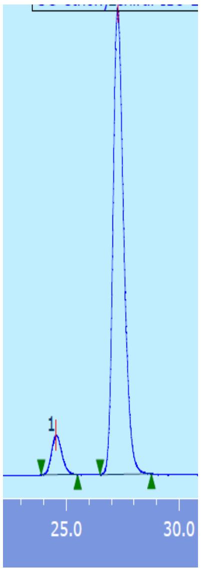

No.	tR (min)	Area (%)	High (%)
1	24.833	50.018	50.086
2	27.583	49.982	49.914

No.	tR (min)	Area (%)	High (%)
1	24.542	7.634	7.805
2	27.258	92.366	92.195

Methyl 2-((difluoromethyl)thio)-2-methyl-3-oxo-3-phenylpropanoate (3h)

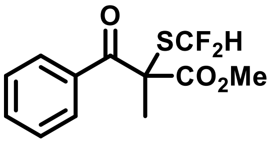

chiral pack OD3, Hexane/*i*PrOH = 99:1, 0.53 mL/min

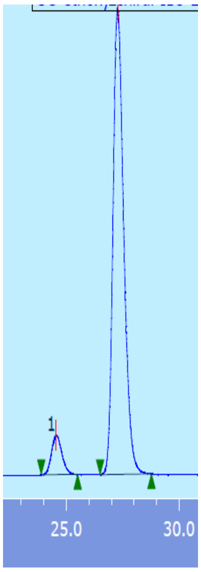

No.	tR (min)	Area (%)	High (%)
1	40.200	43.763	47.481
2	45.317	56.237	52.519
